# Temporal and environmental drivers of fish-community structure in tropical streams from two contrasting regions in India

**DOI:** 10.1371/journal.pone.0227354

**Published:** 2020-04-09

**Authors:** Rubina Mondal, Anuradha Bhat

**Affiliations:** Department of Biological Sciences, Indian Institute of Science Education and Research Kolkata, Mohanpur, West Bengal, India; Universidade Regional Integrada do Alto Uruguai e das Missoes, BRAZIL

## Abstract

Environmental and anthropogenic factors are known to drive fish community structure in aquatic systems across the world. This study investigates fish assemblages in lower order streams across contrasting landscapes in central and eastern India. We documented the species diversity of these monsoon driven lower order streams in the two regions. We also investigated the potential common environmental drivers of richness and diversity and effect of season in these tropical streams. The study was based on seasonal data on abundance of fishes and environmental parameters collected between 2015–2017 from streams in states of Madhya Pradesh and West Bengal. Species diversity were compared across regions and seasons, based on their richness (SR) as well as diversity (Shannon index H'). Drivers of overall richness and diversity were analyzed using multiple linear regression methods, based on best subset selection. Analysis of data revealed high diversity in these streams in both regions. Cyprinidae, Bagridae and Channidae were the most dominant families in both regions. Despite the geographical and local ecological differences across the regions, common environmental parameters were found to influence richness and diversity across the two regions, indicating these as being key drivers of fish community structure. Water flow was a common factor driving both richness and diversity across both regions. Our study revealed a lack of seasonal effect in structuring fish communities in tropical streams. With stream and river ecosystems facing increasing threats due to habitat alterations and water quality degradation in countries such as India, a clear understanding of regional and local drivers of community structure of aquatic fauna is crucial. These results on the role of common environmental factors across ecoregions provides baseline information for understanding their ecological roles and developing management plans for important river basins and fish conservation in future.

## Introduction

With increasing anthropogenic pressure and deteriorating water quality in recent decades, freshwater ecosystems are among the most threatened habitats on earth [1–6]. In recent years, many studies have investigated global and local effects of abiotic and human induced factors on aquatic biodiversity [7–10]. These studies attempt to understand local-regional influences along with larger scale climatic impacts on diversity patterns. Very few studies, however, have explored fish faunal patterns in tropical streams in south-east Asia such as in India, and role of ecological factors and human induced influences on community assemblages. This knowledge is especially relevant now, in the current scenario of increasing anthropogenic changes to freshwater ecosystems [5]. We studied the diversity patterns and spatio-temporal dynamics in fish communities in two contrasting eco-regions located in central and northeastern India, to explore the specific ecological factors, common to these eco-regions that can drive community structure. We used predictive models, based on multiple linear regression, to understand relationships between specific environmental factors and parameters of fish diversity.

As mechanisms underlying community assembly and factors leading to habitat degradation can differ across regions, unanimous causative ecological factors driving diversity and distribution patterns cannot be specified. Studies across various regions point to the importance of different environmental factors such as temperature and climate change [8], [11–13] and physio-chemical factors [14–19]. Over the recent decades, freshwater ecosystems are under greater threat than terrestrial systems [20] and fishes are increasingly being recognized as important indicators of ecosystem health [21], [22]. Along with anthropogenic activities such as dams, pollution and stream modification [23–26], habitat degradation, and invasive species are considered as the leading causes of loss of biodiversity [27–29]. Individual contributions of each of these causative factors need to be assessed to tackle issues of biodiversity decline [30].

Studies to understand influence of local and regional factors driving abundance and richness patterns indicate that these are mainly driven by an interplay between spatial and temporal factors. These observations, however, are dependent on the scale of study, the functional unit (species/ functional traits) studied and level of disturbance in study areas. Studies in freshwater systems have shown the importance of local factors affecting species richness [31], [32] and the role of differing environmental variables at scales of impacted habitats [33] (for example, in Amazonian streams [17] and European streams [34], [35]). Some studies have demonstrated regional factors to act directly or indirectly on local factors, which in turn are stronger determinants of local species assemblages [10]. Simultaneous, standardized methods for collecting information on fish diversity and abundance along with data on local and regional environmental parameters can help understand common or diverging factors that play key roles in patterns of community structure across ecological regions.

Detailed information on freshwater fishes, such as species occurrences and their distributions, are lacking within smaller and medium sized stream habitats in regions of countries like India and China. India is considered among the top 25 countries in terms of endemism [36] and ranks third in Asia in terms of overall diversity of freshwater fishes, with revised estimates of 2,200 fish species in India [37]. Typically, much of the existing literature on fish diversity in the Indian subcontinent focus on major rivers, with few details on diversity patterns in lower order streams. A few studies have investigated diversity and environmental drivers of lower-higher order streams, most of which are in the Himalayan region and Western Ghats which are global biodiversity hotspots [38–41]. Except for isolated studies, data are sparse from many other regions. Most studies on freshwater fish are typically on commercially important species, important from the perspective of the fishing industry. With the increasing global concerns of environmental degradation on aquatic habitats; it is important to understand the role of local and regional factors in shaping community structure in order to develop sound management and conservation plans for tropical aquatic ecosystems within India. Our study explores stream fish communities across two contrasting landscapes to document their species diversity and to understand the role of local and regional environmental drivers that affect their distribution patterns. We investigate the spatial and temporal patterns in freshwater fish communities in lower order streams by asking the following questions: 1) do contrasting landscapes (from within the same broad geographic region have different diversity? 2) Are the key drivers of species richness and diversity common across these landscapes? 3) Are these fish community properties (i.e. richness and diversity) affected by seasonal changes?

Two areas within the Indian subcontinent, one in central India (Madhya Pradesh), and the other in eastern India (northern West Bengal) located in eastern sub-Himalayas were selected for the study. We hypothesize that our study regions would show broadly similar richness and diversity patterns as these landscapes occur within the tropical belt, despite having local level environmental and ecological dissimilarities. Since all sites studied have similar levels of anthropogenic influence, in form of human habitation and agriculture, we do not expect community composition of sites to be differentially affected by disturbance. We also expect these communities to maintain temporal (i.e. seasonal scale) stability as the study regions fall in the tropical belt which is characterized by lack of distinct seasons. Fish diversity across many tropical ecosystems are believed to be determined by some key ecological factors and similar physical and chemical characteristics within streams can affect fish communities. Thus, while overall fish diversity in the two regions are expected to differ, we predict that specific common ecological parameters are likely to drive fish community structure across these two eco-regions.

## Methods

### Ethics statement

No animals were harmed during this study and all individuals were returned to their natural habitats in the field. The study protocol carried out were in accordance with the ethical standards of the institution (Institutional Animal Ethics Committee's (IAEC)), IISER Kolkata and adhered to the guidelines of the Committee for the Purpose of Control and Supervision of Experiments on Animals (CPCSEA), Government of India.

### Study area

**Fig 1:**
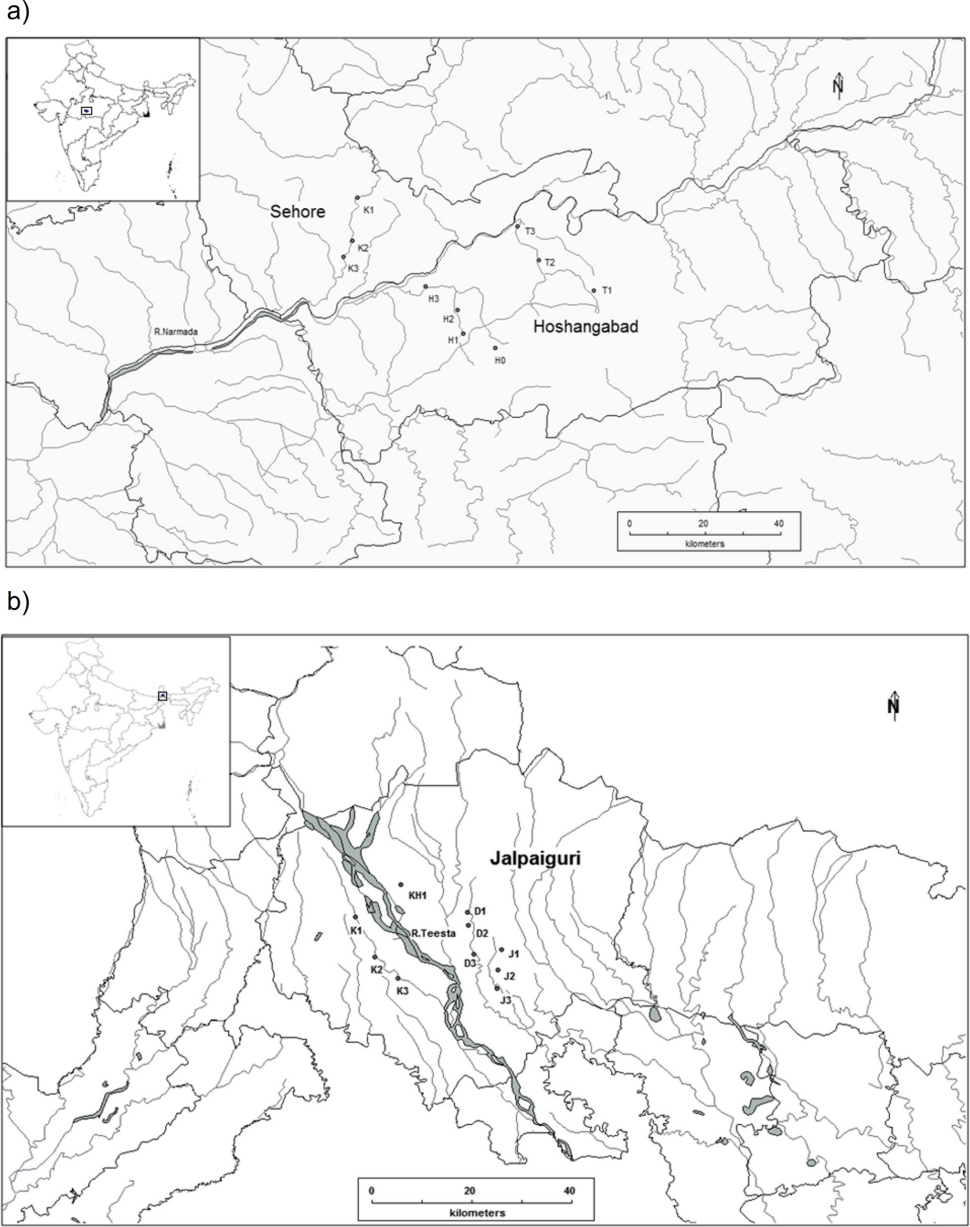
Location of sampling sites in the study regions. a) Sampling sites in Madhya Pradesh, depicted by alphabets for the river name and ascending numbers depicting upstream to downstream. b) Sampling sites in West Bengal, depicted by alphabets for the river names and ascending numbers depicting upstream to downstream. Further details in Tables 1 and 2.

We sampled fish communities in streams across two states, Madhya Pradesh (MP) and West Bengal (WB), in India. Study sites in Madhya Pradesh (in central India) were located between 21.2^0^ N-26.87^0^ N to74.02^0^ E-82.49^0^ E), while the sites in West Bengal (in eastern India) were located between 21.6^0^ N- 27.2^0^ N to 85.8^0^ E- 88.9^0^ E. The two ecological regions are contrasting in terms of landscape, vegetation type, weather patterns, land use, and anthropogenic disturbances. The study sites in MP are located within the Narmada River basin (Fig 1A, Table 1).

**Table 1:** Site names, location and description for Madhya Pradesh.

Site	Stream	Latitude (^0^N)	Longitude (^0^E)	Altitude (m)
H1	Hathed	22.49425	77.72772	319
H2	Hathed	22.52864	77.64906	309
H3	Hathed	22.58531	77.63681	292
H4	Hathed	22.63944	77.55803	294
K1	Kolar	22.84947	77.39153	320
K2	Kolar	22.74861	77.37953	304
K3	Kolar	22.71047	77.35867	295
T1	Tawa	22.63047	77.96694	343
T2	Tawa	22.70286	77.83356	302
T3	Tawa	22.78286	77.78239	292

The Narmada River flows between the rift valley formed by Vindhya and Satpura range which divide India into northern Indo-Gangetic plains and southern Deccan plateau [42]. This area comprises of rocky terrain, dry deciduous forests and fertile plains. The hills have lost most of their original forest cover in the past 15–20 years due to rapid urbanization and industrial growth [43], [44]. The climate is dry and extreme with very little rainfall, restricted to a short monsoon (July-September) season. Fish and ecological data were collected from stream stretches within R.Tawa, R.Hathed and R.Kolar. These streams are characterized by rocky river beds with stream widths ranging from 50m-700m. They are regulated streams facing high anthropogenic pressure due to sand mining, unregulated water use and river bank agriculture [45]. The study sites in West Bengal are located approximately 1,200 km. from the sites in MP. These sites are situated at the foothills of Eastern Himalayas in the Teesta basin (Fig 1B, Table 2).

**Table 2:** Site names, location and description for West Bengal.

Site	Stream	Latitude (^0^N)	Longitude (^0^E)	Altitude (m)
K1	Karala	26.68383	88.57358	114
K2	Karala	26.61172	88.60883	87
K3	Karala	26.57369	88.65	76
KH1	Khulnai	26.74294	88.65614	105
D1	Dharala	26.61775	88.78897	88
D2	Dharala	26.66975	88.77875	90
D3	Dharala	26.69361	88.77714	112
J1	Jarda	26.62639	88.83922	82
J2	Jarda	26.58817	88.83294	101
J3	Jarda	26.55503	88.83122	76

(Sites have been coded with alphabets designating streams and numbers indicating upstream to downstream)

The region is known as the Terai and comprises of rich agricultural alluvial plains with humid climate and heavy rainfall [46]. These streams are narrow (15-50m.), occurring at altitude ranges from 90-100m m.s.l. Sampling was carried out in R. Karala, R. Khulnai, R. Dharala and R. Jarda. The region is well known for its tea plantations and agriculture. Anthropogenic pressures range from forest fragmentation [47], sand mining [48], unplanned river obstruction/diversion and dependence on river water for bathing and washing [49], [50].

## Site selection and data collection

Ten sites, each in West Bengal and Madhya Pradesh were sampled thrice a year during winter (January), pre-monsoon (April-May) and post monsoon (September-October) from 2015 to 2017 (Fig 1A and 1B). Sites were chosen so that they represented the general habitat conditions within the area, could be regularly accessed, and also such that variation in community composition in upstream, midstream and downstream segments can be distinctly characterized. Sites were chosen at a minimum distance of 4–5 kms from each other so as to avoid redundancy due to similarity in geographic conditions.

At each site, fishes were sampled along a 100 m stretch that incorporated the existing microhabitat characteristics within the stream reach [45]. Sampling was conducted for 1.5 to 2 hours at each site, based on previous studies at these sites that have shown saturation of accumulation curves with this effort [45]. Equal sampling effort was maintained across all sites in both study regions. Cast net (mesh size = 10 mm), drag net (mesh size = 1 mm) and gill nets (mesh size = 15 mm) were used for collecting fish samples. Only data on adult fishes were considered for the analysis. Individuals were identified at site [51] or if unidentified, preserved in 4% formalin or 70% alcohol and brought back to the laboratory for later identification. Abundance was noted for each species and fishes were released back into water after identification.

Simultaneously, along with fish sampling, data on local ecological factors characterizing stream properties and its surroundings were collected. This included water quality parameters and stream characteristics- pH, conductivity, total dissolved solids and water temperature using Hanna multi-parameter HI991300 probe; dissolved oxygen using YSI DO meter (YSI55DO); stream width and stream depth using measurement tapes, taking an average of 3 measurements at each site; water velocity (by measuring velocity of floating cork); and altitude using GARMIN trex 30 (Table 3). Riparian vegetation and surrounding land use pattern were also noted.

**Table 3:** Details of physico-chemical and habitat parameters measured at sampling locations in MP and WB- Mean, minimum (Min.), maximum (Max.) and standard deviations (S.D.) are given in the table below.

	Mean		Min.		Max.		S.D.	
Factor	WB	MP	WB	MP	WB	MP	WB	MP
Physico-chemical parameters								
pH	6.73	7.95	5.68	7.08	7.86	9.08	0.50	0.48
Conductivity (μS/cm)	63.17	408.44	31.00	125.00	119.00	780.00	15.82	156.80
Total dissolved solutes (ppm)	31.54	203.03	15.00	64.00	59.00	389.00	7.95	78.26
Dissolved oxygen (mg/l)	7.96	8.59	4.04	2.51	11.24	16.96	1.26	2.52
Water temperature (^0^C)	26.18	25.38	17.30	16.35	34.50	36.00	3.93	4.34
Habitat parameters								
Stream Width (m)	17.51	96.54	3.50	0.60	75.00	1000.00	11.56	189.69
Stream Depth (m)	0.82	2.94	0.10	0.10	3.95	40.00	0.65	6.64
Water Velocity (m/s)	0.61	0.19	0.00	0.00	25.20	1.23	2.63	0.29
Altitude (m)	92.87	309.10	76.00	292	114.00	384.00	13.23	20.05
Rainfall (cm)	244.80	44.11	1.90	0.20	679.20	219.10	252.92	65.40

(Min = Minimum recorded value; Max = Maximum recorded value; S.D. = Standard deviation)

### Data analysis

Expected species richness was calculated using species accumulation curves using the method of rarefaction. Alpha diversity using Shannon-Wiener index (H') of diversity, and species richness (SR) were calculated and analyzed separately for both regions [52]. Conservation status of sampled species were obtained from IUCN red list of threatened species. Species were classified into their respective families and analyzed for numerical dominance of families. Most abundant families and percentage of shared species were compared across both regions. Both H' and SR were checked for normality using Shapiro-Wilk’s test for normality. The data were found to be normally distributed for both MP (H'_MP_: W = 0.95, p-value = 0.15; SR_MP_: W = 0.97, p-value = 0.46) and WB (H'_WB_: W = 0.95, p-value = 0.14; SR_WB_: W = 0.97, p-value = 0.54). Diversity (Shannon index, H') and species richness (SR) were first compared for the entire dataset and across seasons between both regions using Welch's unequal variances t-tests. Season-wise H' and SR were then compared between the regions to rank them over diversity and abundance using pairwise t-test and p-values were adjusted using Bonferroni correction method for multiple paired comparisons. Correlation among the environmental factors and richness and diversity was checked using Pearson correlation. Hierarchical cluster analysis, using Bray-Curtis distances and complete linkage algorithm, were checked for seasonal patterns among communities within a region. ANOVA was performed on seasonal richness and diversity within each region followed by a post-hoc analysis with Tukey’s test.

Linear modeling was then employed for developing predictive models for species richness and diversity, based on selected environmental parameters. Ordinary least square regression models were built with Shannon index and species richness separately as response variables and the environmental and habitat characteristics as predictor variables. Coefficient of variation for the environmental variables were calculated for each site for each season (3 samples per site per season). Correlation amongst variables was checked using variance inflation factor (VIF) on the linear model containing all variables [53], [54]. Variables having highest values were removed sequentially till VIF did not exceed 5 for the rest of the variables. These explanatory variables then formed a global model which was subjected to stepwise regression. The global model comprised of 12 variables in WB and 13 in MP. In general, the stepwise regression model is constructed by sequentially adding or deleting one independent variable. Here, bidirectional approach is applied where the model is built by combining the techniques of the forward selection with backward eliminations till a model is reached whose Akaike Information Criterion (AIC) and residual deviance is the least [55]. The *ols* package in R creates all possible subsets for the given variables by stepwise regression. They were ranked according to their explanatory power (Adjusted R^2^) and AIC values. The most parsimonious model was chosen based on AIC values as well as biological rationality. Potential models were compared using ANOVA against the model with the least AIC. We also performed redundancy analysis (RDA), with all environmental factors following stepwise regression on both richness and abundance, on Hellinger transformed abundance data. VIF was checked on models so as not to increase above 5. Significance of overall models and individual terms were assessed using *anova*.*cca* (*vegan* package in R).

All statistical tests were conducted in R (R Core Team 2015, ver 3.3.2, 2016) using *vegan* [56] and *olsrr* [57] packages.

## Results

### Alpha diversity patterns

**Fig 2:**
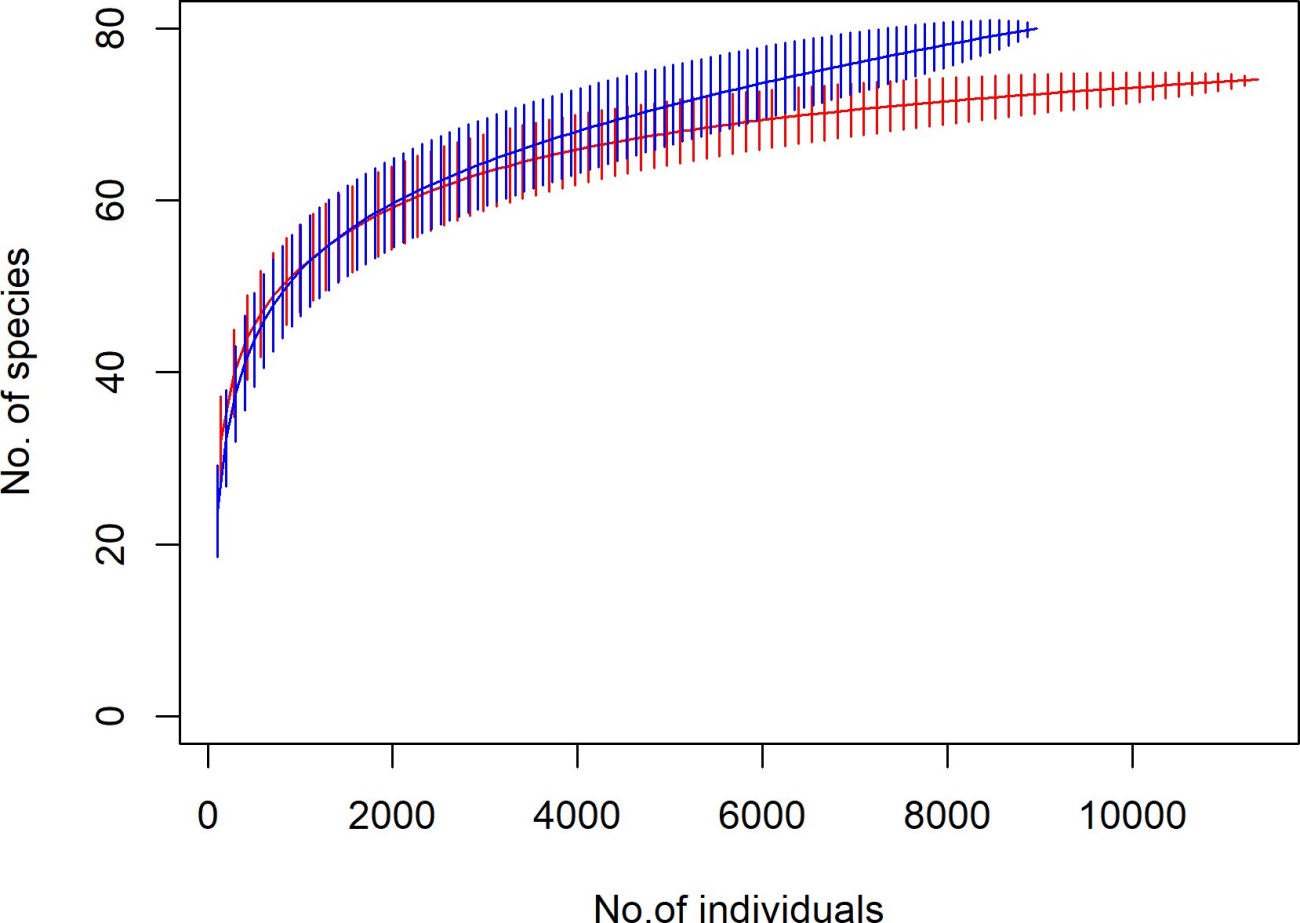
Comparative richness in fish species of Madhya Pradesh and West Bengal. Species accumulation curve (method = rarefaction) showing differences in total number of species per individuals sampled for MP (in red) and WB (in blue).

A total of 20,186 individuals was sampled from both regions (N_MP_ = 11,223, N_WB_ = 8,963). Species accumulation curves by rarefaction showed West Bengal to be more speciose (total species richness = 71) than Madhya Pradesh (total species richness = 69) (Fig 2, Tables 4 and 5, S1 Table). Data from IUCN red list revealed 4 species in WB and 6 species in MP which fall under either near threatened, vulnerable or endangered category (S1 Table).

**Table 4:** Details of diversity (H') and richness (SR) for sampling locations during seasonal sampling periods in Madhya Pradesh.

	Winter		Pre monsoon		Post monsoon	
Site	H'	SR	H'	SR	H'	SR
H1	2.30	21	2.18	17	2.658	27
H2	2.36	22	2.49	25	2.334	28
H3	1.95	27	2.47	32	2.287	24
H4	1.99	18	2.54	30	2.236	18
K1	2.41	23	2.52	22	2.037	21
K2	2.33	19	2.74	27	2.367	25
K3	2.28	22	2.30	25	2.256	24
T1	1.48	18	2.36	21	2.445	25
T2	1.81	21	1.85	15	2.028	18
T3	1.72	25	2.19	18	2.486	22

**Table 5:** Details of diversity (H') and richness (SR) for sampling locations during seasonal sampling periods in West Bengal.

	Winter		Pre monsoon		Post monsoon	
Site	H'	SR	H'	SR	H'	SR
K1	1.49	19	1.95	19	2.23	15
K2	2.36	22	2.31	23	2.20	20
K3	1.46	20	2.62	27	2.38	27
KH1	2.12	18	2.29	24	1.85	19
D1	1.64	19	2.50	27	2.78	29
D2	1.58	17	2.14	15	2.57	22
D3	1.10	20	2.49	25	1.39	23
J1	1.80	17	2.41	19	1.12	14
J2	2.06	19	2.26	23	2.65	21
J3	1.80	26	2.31	22	1.33	27

Within each region, ANOVA revealed no effect of season on species richness in MP or WB (p_MP_ = 0.58, p_WB_ = 0.34). Seasonal differences in diversity was observed between winter and pre-monsoon in both regions (p_MP_ = 0.04, p_WB_ = 0.001). Cluster dendrograms revealed no clear seasonal patterns in either WB or MP (S1 Fig).

**Fig 3:**
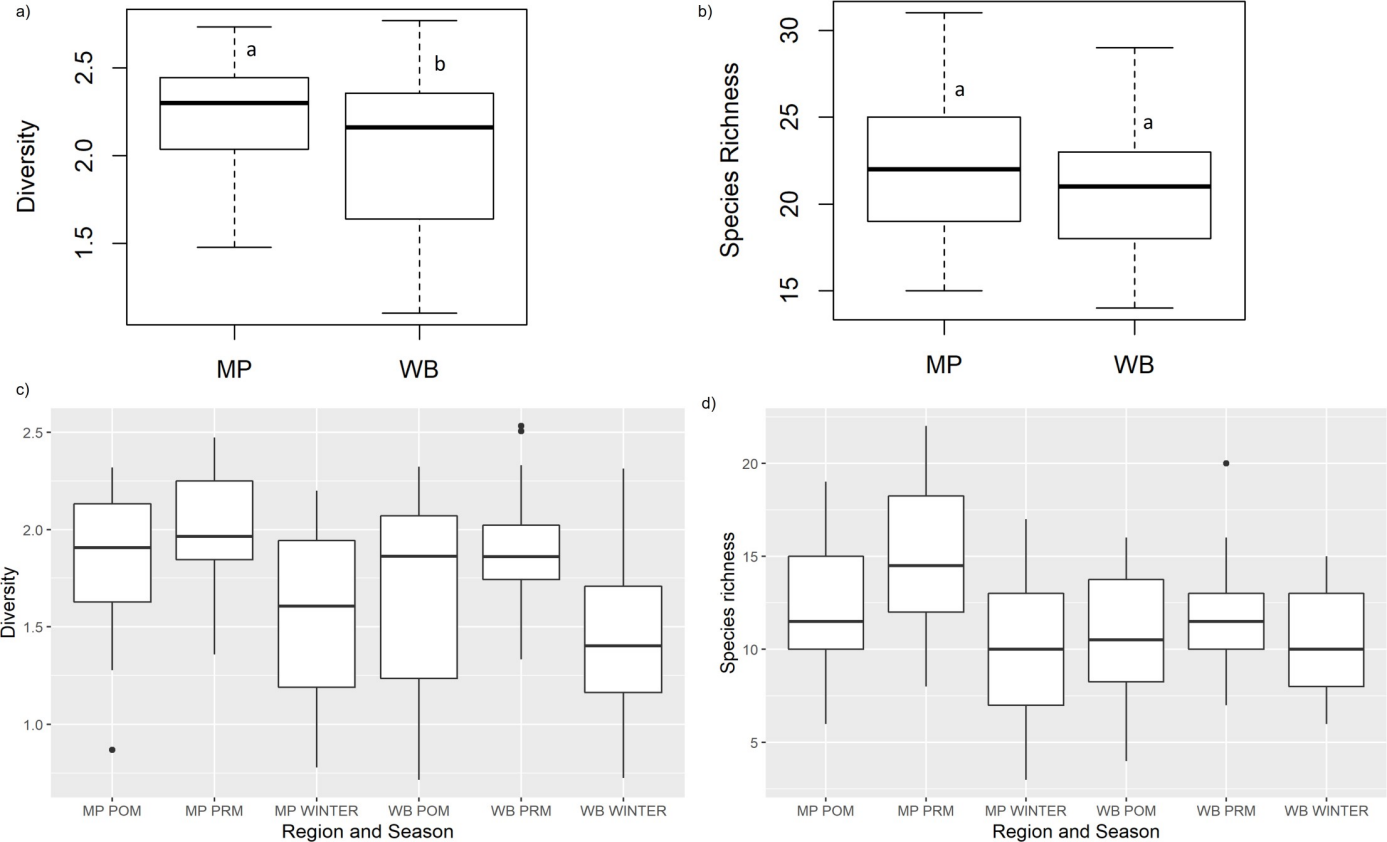
Comparison of combined and temporal patterns in diversity and richness across MP and WB. a) Comparison of overall diversity between MP and WB show significant difference (t = -2.21, p-value = 0.03) but not in b) overall species richness (t = -1.42, p-value = 0.16). Temporal patterns in c) seasonal diversity (H') and d) seasonal species richness (SR) in Madhya Pradesh and West Bengal reveal pre monsoon to have the most abundant and diverse community. Lines, boxes, and whiskers depict medians, interquartile ranges (IQR), and 1.5xIQR of the estimated H' and SR, respectively.

Welch's unequal variances t-tests show significant difference in diversity (t = -2.21, df = 48.19, p-value = 0.03, Fig 3A) but not in species richness (t = -1.42, df = 57.60, p-value = 0.16, Fig 3B) between MP and WB (Tables 4 and 5).

Comparison between MP and WB revealed pre- monsoon season as highest in terms of richness and diversity for both MP and WB (SR_MP,PRM_ = 15.6,H'_MP,PRM_ = 2.33; SR_WB,PRM_ = 12.47, H'_WB,PRM_ = 3.06). It was followed by post monsoon (SR_MP,POM_ = 12.4, H'_MP,POM_ = 2.11) and winter (SR_MP,WIN_ = 10.36, H'_MP,WIN_ = 2.02) in MP. In WB, abundance decreased in the order of winter to post monsoon (H'_WB,WIN_ = 3.04, H'_WB,POM_ = 2.4) and vice-versa for SR (SR_WB,POM_ = 11.16, SR_WB,WIN_ = 10.36) (Fig 3C and 3D).

Species richness (SR) during pre-monsoon in MP was significantly greater than post monsoon samples and was significantly different from species richness across all seasons in WB (p<0.003). Winter and pre-monsoon samples were significantly different in terms of diversity (H') in both MP and WB (p = <0.003) (S2 Table).

Out of total 94 species, 44 species (46.8%) are shared between these two regions. 50 unique (53.2%) species were present in both regions combined.

#### Family distribution patterns

**Fig 4:**
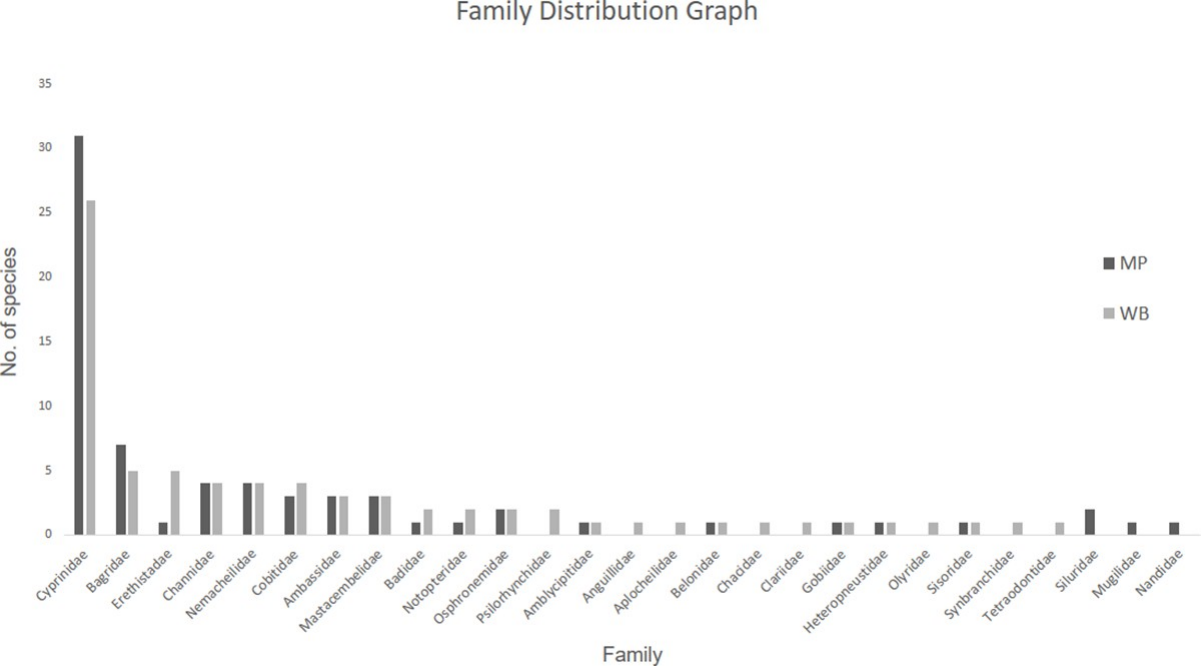
Comparison of fish family distribution across Madhya Pradesh and West Bengal. Among the 24 fish families in WB and 19 families in MP, Cyprinidae was the most dominant family (MP = 45%, WB = 34.25%) followed by Bagridae (MP = 10.14%, WB = 6.85%) and Channidae and Nemacheilidae (MP = 5.79%, WB = 5.48%) in both regions.

Fishes collected in West Bengal belonged to 24 families whereas fishes of Madhya Pradesh belonged to 19 families (Fig 4). Cyprinidae (MP = 45%, WB = 34.25%), Bagridae (MP = 10.14%, WB = 6.85%) followed by Channidae and Nemacheilidae (MP = 5.79%, WB = 5.48%) were the most dominant families in both regions. Cyprinidae comprised of 31 species and 25 species in MP and WB, respectively. Bagridae was represented by 7 and 5 species in MP and WB respectively, while Channidae and Nemacheilidae had 4 species each in both the regions.

#### Environmental drivers of richness and abundance within each eco-region

Univariate correlations (Pearson’s correlations) between community indices and environmental factors revealed some significant relationships. Water velocity was found to be significantly correlated to H' (*r* = -0.37, p = 0.04) and SR (*r* = -0.39, p = 0.03) in WB. Conductivity was correlated to H' (*r* = 0.4, p = 0.02) and SR (*r* = 0.46, p = 0.01) in MP. Total dissolved solids (*r* = 0.46, p = 0.01) and water temperature (*r* = 0.39, p = 0.03) were also found to be correlated to H' in MP (S3 Table).

The selected predictive model (using stepwise regression and model selection based on parsimony) for SR included variation in conductivity, water temperature and stream depth, along with water velocity and altitude (Adj.R^2^ = 0.62) for WB. All factors, variations in conductivity (Estimate = 35.55, p = <<0.01), stream depth (Estimate = -6.94, p = 0.006), water temperature (Estimate = - 41.37, p = 0.004) and water velocity (Estimate = -1.38, p = <<0.01) and altitude (Estimate = -0.13, p = 0.003) showed significant effect (Table 6). H' was found to be significantly affected by dissolved oxygen (Estimate = -0.22, p = 0.03) and water velocity (Estimate = -0.11, p = 0.05). While variation in stream depth was also included in the model, it was not significant (Table 6).

**Table 6:** Summary of final models chosen for explaining Species Richness (SR) and Shannon diversity (H') for both study regions. Significant factors, i.e. p≤0.05 are shown in bold.

**West Bengal**				
**Species Richness** (SR) ~ Conductivity.cv + Temperature.cv + Stream depth.cv + Water velocity + Altitude				
AIC		Adj. R^2^	p value	
147.76		0.59	**<<0.001**	
Variable	Estimate	Std. Error	p value	
Conductivity.cv	35.55	9.01	**0.001**	
Temperature.cv	-41.37	12.89	**0.004**	
Stream depth.cv	-6.94	2.32	**0.006**	
Water velocity	-1.38	0.32	**<<0.001**	
Altitude	-0.13	0.04	**0.003**	
**Diversity (H’) ~** Dissolved oxygen + Stream depth.cv + Water velocity				
AIC		Adj. R^2^	p value	
38.06		0.23	**0.002**	
Variable	Estimate	Std. Error	p value	
Dissolved oxygen	-0.22	0.09	**0.027**	
Stream depth.cv	-0.11	0.05	**0.048**	
Water velocity	-0.55	0.38	0.158	
**Madhya Pradesh**				
**Species Richness** (SR) ~ pH + Conductivity + Dissolved oxygen + Water velocity				
AIC		Adj. R^2^	p value	
165.15		0.32	**0.01**	
Variable	Estimate	Std. Error	p value	
pH	8.82	2.97	**0.007**	
Conductivity	0.02	0.01	**0.004**	
Dissolved oxygen	-0.60	0.39	0.132	
Water velocity	5.28	3.39	0.132	
**Diversity** (H') ~ Conductivity + Total dissolved solids.cv + Temperature + Rainfall.cv				
AIC		Adj. R^2^	p value	
2.25		0.38	**0.002**	
Variable	Estimate	Std. Error	p value	
Conductivity	0.00	0.00	**0.037**	
Total dissolved solids.cv	1.87	0.65	**0.008**	
Temperature	0.03	0.01	**0.021**	
Rainfall.cv	0.54	0.28	0.068	

(H' = Shannon Wiener Diversity Index; SR = Species Richness; AIC = Akaike Information Criteria; Adj. R^2^ = Adjusted R^2^)

The selected predictive model (using stepwise regression and model selection based on parsimony) for SR included pH, conductivity, dissolved oxygen and water velocity and variation in rainfall (Adj.R^2^ = 0.32) for MP. Among these, pH (Estimate = 8.82, p = <0.01) and conductivity (Estimate = 0.02, p = <0.01) were significant response variables (Table 6). Final model for H' included variations in total dissolved solids and rainfall, along with conductivity and water temperature (Adj. R^2^ = 0.38). Out of these, significant effect was seen for variation in total dissolved solids (Estimate = 1.87, p = <<0.01), conductivity (Estimate = 0.001, p = 0.04) and temperature (Estimate = 0.03, p = 0.02) (Table 6).

**Fig 5:**
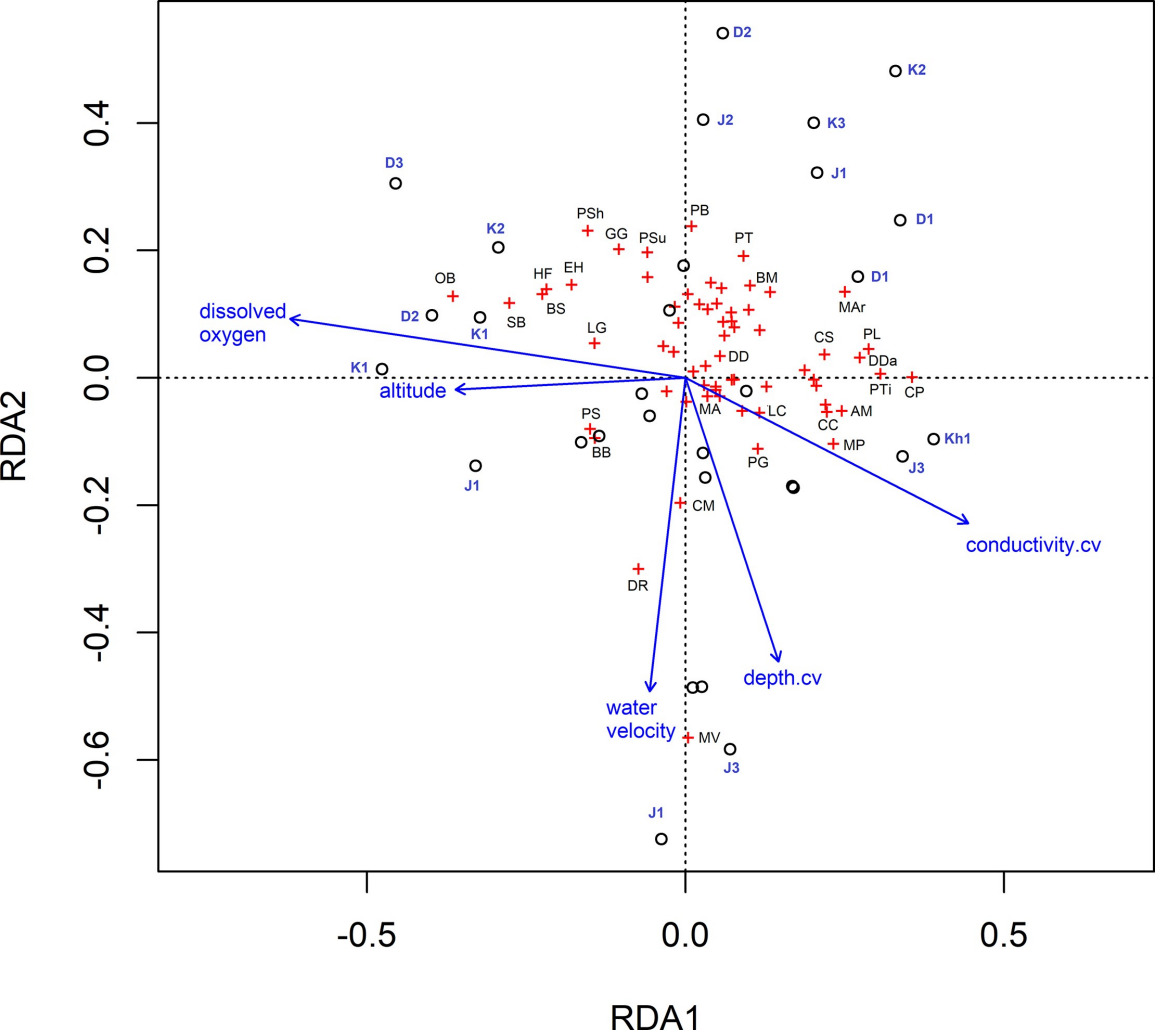
Redundancy analysis of 30 fish assemblages and environmental factors in West Bengal. Only the factors which were chosen after forward selection related to alpha diversity are shown on the plot above. Individual assemblages are denoted by black circles accompanied by site name in bold blue text (details in Table 2) and species by red crosses accompanied with initials of species names in black. Vector arrows are labelled with the corresponding environmental factor, and the length of the arrows indicates the relative strength of the relationship between that factor and assemblage structure. Legend for species names: *OB = Ompok bimaculatus; SB = Schistura beavani; BS = Barilius shacra; HF = Heteropneustes fossilis; EH = Erethistes hara; LG = Lepidochephalichthys guntea; PSh = Pseudolaguvia shawi; GG = Glossogobius giuris; PSu = Psilorhynchus sucatio; PB = Paracanthocobitis botia; PT = Puntius terio; BM = Barilius modestus; MAr = Mastacembelus armatus; CS = Channa striata; PL = Parambassis lala; DDa = Danio dangila; PTi = Pethia tictio; AM = Amblypharymgodon mola; CC = Chitala chitala; MP = Macrognathus pancalus; CP = Channa punctata; LC = Leiodon cutcutia; MA = Macrognathus aculeatus; PG = Pethis gelius; CM = Clarias magur; DR = Danio rerio; BB = Barilius bengalensis; PS = Puntius sophore*.

**Fig 6:**
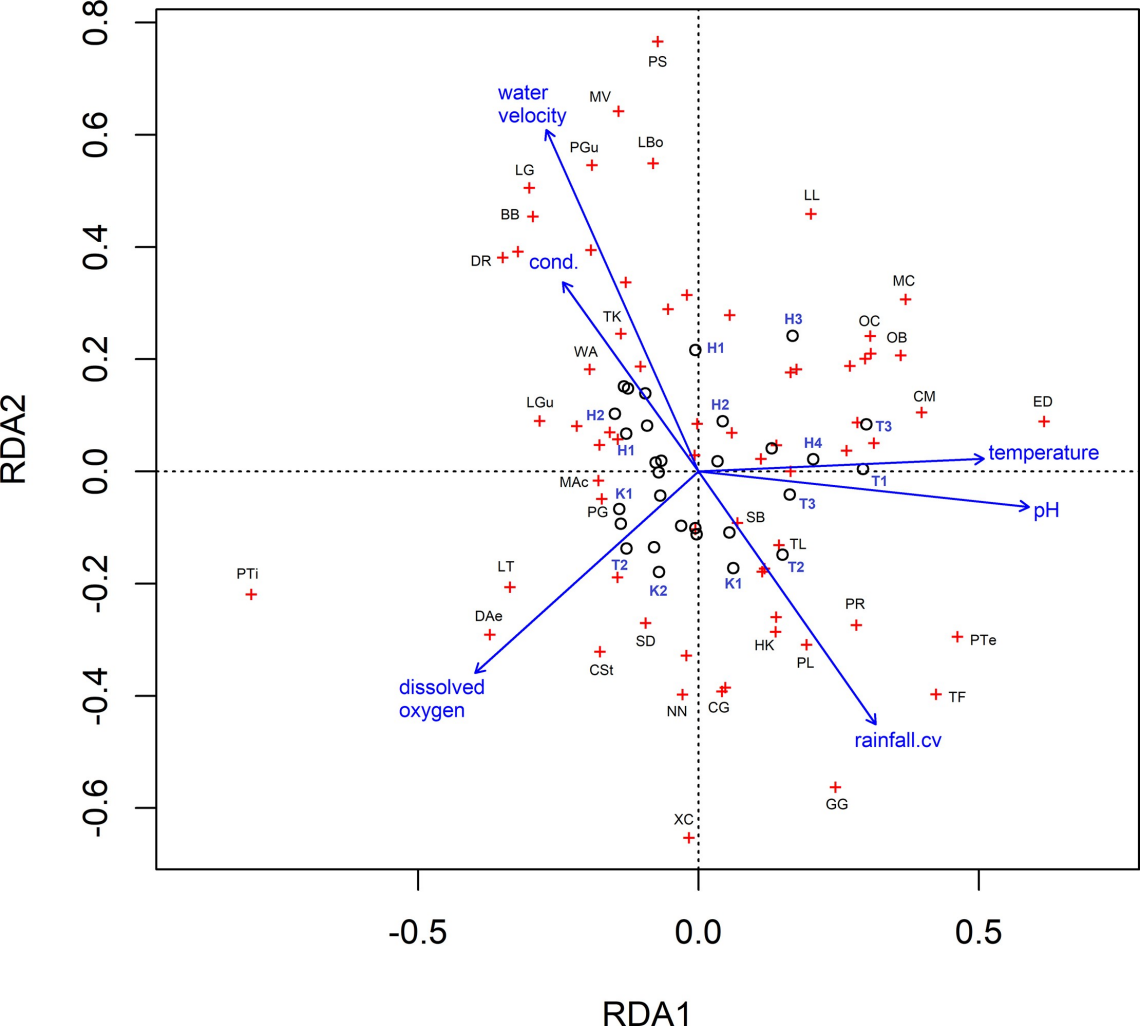
Redundancy analysis of 30 fish assemblages and environmental factors in Madhya Pradesh. Only the factors which were chosen after forward selection related to alpha diversity are shown on the plot above. Individual assemblages are denoted by black circles accompanied by site name in bold blue text (details in Table 1) and species by red crosses accompanied with initials of species names in black. Vector arrows are labelled with the corresponding environmental factor, and the length of the arrows indicates the relative strength of the relationship between that factor and assemblage structure. Legend for species names: *LG- Labeo gonius; BB- Barilius bendelensis; DR- Danio rerio; TK- Tor khudree; WA- Wallago attu; LGu- Lepidochephalichthys guntea; MAc- Macrognathus aculeatus; PG- Pethia gelius; LT- Lepidocephalichthys thermalis; DAe- Devario aequipinnatus; CSt- Channa striata; SD- Schistura denisoni; NN- Nandus nandus; CG- Channa gachua; XC- Xenentodon cancila; GG- Glossogobius giuris; HK- Hypselobarbus kurali; PL- Parambassis lala; TF- Trichogaster fasciatus; PTe- Puntius terio; PR- Parambassis ranga; TL- Trichogaster lalia; SB- Schistura beavani; ED- Esomus danrica; CM- Channa marulius; OB- Ompok bimaculatus; MC- Mystus cavasius; LL- Laubuka laubuca; PS- Puntius sophore; MV- Mystus vittatus; PGu- Pethia guganio; LBo- Labeo boggut*.

RDA models were significant for both MP and WB (WB: F = 1.46, p = 0.02; MP: F = 2.24, p = 0.001) (Tables 7 and 8). In the model for WB, 23.4% of total variation was explained by the constrained axes. The first three RDA axes explain 87.6% of this variation. Variation in conductivity (F = 1.8, p = 0.05) and dissolved oxygen (F = 1.97, p = 0.04) showed highest loadings on the first axis, while water velocity and variation in depth showed the highest loading on the second axis. In the RDA model for MP, pH, conductivity, dissolved oxygen, water velocity, temperature and variation in rainfall were used as constraints. The model explained 36.85% of the overall variation and the first three axes represented 73.72% of this variation. All the environmental variables were significant (p<0.05). First RDA axis showed maximum loading for pH and temperature while water velocity and variation in rainfall showed highest loading in the second axis (Figs 5 and 6).

**Table 7:** Eigenvalues and proportion explained by the first three axes of RDA along with axis loadings of environmental variables on first two RDA axes along with their significance based on permutation tests (number of permutations = 999) in WB. Values lesser than 0.05 (in bold) are considered as significant explanatory factors for the community structure.

	RDA1	RDA2	RDA3	
Eigenvalue	0.05	0.02	0.01	
Proportion explained	0.51	0.22	0.14	
Cumulative proportion	0.51	0.73	0.88	
Factor	Axis 1	Axis 2	F	p-value
**Conductivity.cv**	0.59	-0.37	1.8	**0.04**
Stream depth.cv	0.19	-0.73	0.98	0.44
Water velocity	-0.07	-0.80	1.34	0.15
Altitude	-0.48	-0.03	1.22	0.25
**Dissolved oxygen**	-0.82	0.15	1.97	**0.04**

**Table 8:** Eigenvalues and proportion explained by the first three axes of RDA along with axis loadings of environmental variables on first two RDA axes along with their significance based on permutation tests (number of permutations = 999) in MP. Values lesser than 0.05 (in bold) are considered as significant explanatory factors for the community structure.

	RDA1	RDA2	RDA3	
Eigenvalue	0.05	0.04	0.02	
Proportion explained	0.34	0.24	0.16	
Cumulative proportion	0.34	0.58	0.74	
Factor	Axis 1	Axis 2	F	p-value
**pH**	0.50	-0.06	2.14	**0.005**
**Conductivity**	-0.21	0.34	1.76	**0.023**
**Dissolved oxygen**	-0.34	-0.37	2.39	**0.001**
**Water velocity**	-0.23	0.62	2.70	**0.001**
**Rainfall.cv**	0.27	-0.46	1.88	**0.009**
**Temperature**	0.44	0.02	2.54	**0.001**

## Discussion

The main objective of our study was to compare diversity and richness across two eco-regions of the Indian subcontinent and to tease apart the main drivers of diversity in these two regions. Our results revealed high species diversity within these lower order streams highlighting the importance of studying these systems as much as the higher order streams and major rivers. Based on a 3-year collection of fish samples across the two eco-regions, the study allowed for investigation of differences in diversity across seasons (temporal) and regions (spatial). While overall diversity and abundances in the two regions were found to be comparable, ecological factors such as conductivity, dissolved oxygen and water velocity were found to be microhabitat level common ecological factors driving richness and diversity patterns within these regions. Besides these factors, other factors were found to specifically determine patterns within each of these regions.

### Diversity patterns within eco-regions

In terms of their fish fauna, our study revealed comparable overall richness and diversity across the two regions (with species richness of 71 and 69 for WB and MP, respectively). Saturating rarefaction curves indicated adequate sampling during the 3 years of the study, as also seen in similar studies in the Indian subcontinent [40], [45]. With around 70 species in both regions, our study showed a reasonably high diversity of freshwater fishes for even lower order reaches, comparable with somewhat larger sized rivers across India [45], [58–60]. Despite being more than 1,200 km. from each other, fish communities in these regions show similar patterns in terms of pre-monsoon, being the season for maximum richness and abundance, and similar common dominant families. Pre-monsoon was found to be the most diverse and abundant in terms of numbers, possibly because of reduced niche availability and consequent high density of individuals due to prevalent environmental conditions. The streams often get reduced to isolated pools and most species become aggregated in these habitats. This pattern has been observed in other tropical communities too [61], [62]. Although MP and WB showed identical patterns for species richness, in terms of abundance, WB showed a difference, with winter as the second most diverse season followed by post monsoon. This maybe because of the extremely high amount of rainfall that it receives, leading to floods and complete removal of macrophytes and detrital layer in the streams. With uniform microhabitats and clear waters, there is a lack of shelter, food resources and niches for fishes to thrive.

Cyprinidae is the most dominant family in stream fish assemblages across south-east Asia, as previously shown in many studies in similar environmental conditions [63–65]). Most species in this family are herbivores and planktivores. The other families such as Channidae, Bagridae and Nemacheilidae mostly consist of piscivores, omnivores or detritivores [51]. Feeding ecology of several species from these regions is unclear and more studies are required on diet of these fishes in their native habitats.

### Environmental drivers of richness and diversity

It is well understood that environmental factors at local scales and at regional scales affect fish communities [7], [33], [66]. Our models were based on best fit and biological rationalizing. As expected, our models revealed that several factors together can predict richness and diversity in these two regions. Local physical factors (stream width, stream depth, water temperature and their respective seasonal variations) were found to be more influential than local chemical factors or regional factors in shaping community structure in both regions in terms of richness and abundance. However, regional factors such as rainfall and local chemical factors such as seasonal variation in conductivity and total dissolved solids do explain a part of the variation. Certain factors such as pH, dissolved oxygen and water velocity were found to significantly predict richness and diversity for both regions. Increase in stream depth and width offer more space for individuals and provide more variation in niches, resulting in higher diversity in accordance with species area relationship [67]. Studies have shown habitat volume [68] and habitat size and diversity [67] to have a positive relationship with species richness and abundance. Larger and diverse habitats would facilitate more resource availability and reduced competition [69] along with niche diversification. In MP, pH, dissolved oxygen, water velocity and variation in rainfall were significant drivers for both species richness and diversity compared to water temperature and water velocity in WB. In WB, water velocity was found to be a common factor affecting both diversity and richness. Water flow is a well-recognized factor in shaping aquatic communities [70], [71] and wider, slower flowing downstream habitats generally tend to have higher diversity and richness, along with functional trait diversity, than transient upstream habitats [72]. Dams can obstruct natural flow and can severely impede migration of fish to upstream locations and can alter or fragment natural habitats [73], [74].Water quality parameters like conductivity [75–76] and total dissolved solids [77] are often found to negatively alter water quality conditions and allow for tolerant species to survive. Usually associated with ecological degradation and anthropogenic disturbance [78], [79], total dissolved solids and conductivity have been shown to reduce richness and diversity in Amazonian streams [80] and in streams in eastern India [81] and in Western Ghats [82]. Some studies have also noted the contribution of increased nutrient content in the water to increased primary productivity and persistence of periphyton feeding fishes [83], excessive algal growth and increased sediment settlement [84]. In our study regions, we expect agricultural leaching to be a major factor driving conductivity and dissolved solids in these streams. Indeed, higher amount of aquatic plant and algal growth was observed in MP compared to WB. In MP, sites were often associated alongside large agricultural farms where different crops are grown throughout the year. In WB, however, agricultural leaching is probably less as all sites traversed through permanent tea plantations. Streams in WB were less murky and relatively had lower aquatic vegetation than those in MP. Several other microhabitat and regional factors such as pH, temperature [85], dissolved oxygen [86], rainfall and altitude [41], [87] are known to affect fish assemblage structure. Our selected predictive models for species richness as well as Shannon diversity included electrical conductivity, dissolved oxygen, temperature along with water velocity stream depth and rainfall as significant factors. RDAs show similar major factors in determining community structure. In WB, variation in conductivity and dissolved oxygen are the major drivers of community structure (Fig 5). Fish species such as carps *Opsarius barna* and *Barilius shacra*, predatory fish such as *Heteropneustes fossilis*, and loaches such as *Schistura beavani* and *Erethistis hara* are influenced by increasing strength of dissolved oxygen. Very high variation in conductivity does not support species presence. At medium levels of variation in conductivity and lower dissolved oxygen levels, Mastacembelidae species such as *Macrognathus aculeatus* and *M*. *pancalus* are found. Other species include commercially important fishes such as *Chitala chitala* and *Amblygopharyngodon mola*. In MP, known carnivores such as *Channa punctata*. *C*. *marulius* and *Ompok bimaculatus* are highly driven by higher temperature (Fig 6). Although pH was significant, no fishes were associated with very high or very low pH. Variation in rainfall positively affect glassfish species such as *Parambassis lala* and *P*. *ranga*, and carp species, such as *Puntius terio* and *Hypselobarbus kurali*. Vectors representing variation in rainfall and water velocity are diametrically opposite. Endangered species like *Tor khudree* and *Wallago attu*, along with species such as Indian torrent catfish *A*.*mangois* are associated with high conductivity. Dissolved oxygen affects giant Danio *Devario aequipinnatus*, loaches like *Lepidocephalichthys thermalis*, and commercially important carp such as *Labeo rohita*. Carps like *L*. *boggut* and *L*. *gonius*, striped dwarf catfish *Mystus vittatus* and other small sized fishes such as *Pethia guganio* and *Puntius sophore* are found in fast flowing conditions. Thus, our findings support previous studies where local and regional factors has been shown to be influential in determining diversity patterns [8], [88]. Our findings suggest that local physical microhabitat conditions have a greater role than large-scale factors in shaping community diversity.

### Temporal patterns in richness and abundance

Lack of seasonal variation maybe a result of these two regions lying in a tropical zone where seasons are not as distinct as in temperate regions. In tropical countries, occurrence of clear seasonal temporal patterns seems to be inconsistent across studies and thus needs more in-depth investigation [61], [89], [90]. For this, long-term datasets are required to reveal definite temporal patterns and for understanding their underlying mechanisms [91]. Although our study regions have contrasting geography, aerial environment, stream morphology and water chemistry, annual ranges for most parameters were quite similar. Every season transitions rapidly into another with no extreme conditions affecting either region. Annual ranges in climatic conditions were comparable across these two regions. Our study investigated numerical abundance and richness and the associated differences across seasons. This study did not consider compositional differences that could occur across seasons. Further analyses to understand changes in beta diversity would reveal the role of turnover or nestedness within such communities. Thus, combined investigations of alpha and beta diversity would help in revealing underlying processes that determine community structure and compositions across these regions.

India’s recent water policy in river linking and large scale inter basin water transfer may have serious implications for biodiversity and homogenization of diversity in fishes [92], [93]. Furthermore, lack of exotic and introduced species in our study areas may be indicative of relatively lesser use of these streams for aquaculture. Although annual ranges in environmental parameters were similar, our study sites were quite different in terms of climatic conditions, vegetation and land use type (Table 3). Study sites in northern West Bengal lie at the foothills of the eastern Trans-Himalayan region. These streams drain into R. Teesta which is a sub basin under the larger drainage area of lower R. Brahmaputra comprising of 83 watersheds. Teesta has a drainage area of 12,540 sq. km of which 3,017 sq. km. lie in north Bengal. A large number of irrigation and hydroelectric projects are built on Teesta which serve the entire region [94]. This area is characterized by glacial and rain fed lesser order montane streams. These streams typically have short flowing distances before joining other streams. Vegetation in this region is mostly deciduous mixed with tropical moist evergreen type. Being generally quite densely populated, these streams are under extensive pressure from anthropogenic activities such as stream modification/alteration, waste disposal and unregulated channelization for agriculture.

In contrast, our study area in the upper and middle Narmada basin falls in the eastern part of the central India in the state of Madhya Pradesh. This basin is bounded by the ranges of Vindhyas in the north, Satpura in the south, Maikala in the east and Arabian Sea in the west. Covering an area of 98,796 sq.km, it comprises of 3 sub-basins and 150 watersheds. The geology of the basin around our study sites comprises of basaltic escarpment and hard sandstones with alternate layers of shale. The climate is humid and tropical with four seasons. Dominant land type use is for agriculture (56.9% of the total area) followed by forest lands (32.9%). Rivers in this basin (including rivers Tawa and Kolar) are being used for irrigation and hydroelectric projects [94].

In conclusion, our results suggest that while season might not have a significant effect on either richness or diversity of fish communities in lower order tropical streams (similar to the streams that were studied here) at the spatial scale, local environmental factors are likely to have a greater role in shaping these communities than regional factors. In the light of rapid urbanization and climate change, understanding the underlying ecological processes will help us in better formulating protection and conservation action plans. Studies need to focus on lower order streams as much as major rivers as these streams not only act as contributing sources of biodiversity but also provide connectivity across water bodies.

## Supporting information

S1 Table Consolidated list of species collected from the study regions. Family-wise list of species in Madhya Pradesh and West Bengal. ‘Pr.’ indicates that the species is present in the region.
(DOCX)

S2 Table Pairwise comparisons using t-tests ngalh and seasonal variation in rms of diversitys in ng upstrem to test for seasonal variation. Comparisons across seasons, in Species Richness (SR) and Shannon Wiener diversity (H') among seasons in Madhya Pradesh (MP) and West Bengal (WB). Given below are the t-statistic for pairwise comparisons. Values shown in bold are statistically significant. (p-value adjustment method = Bonferroni; p<0.003 was considered statistically significant).
(DOCX)

S3 Table Correlation tables showing Pearson’s correlation coefficient (r) and its significance for species richness and diversity in Madhya Pradesh and West Bengal (p<0.05 are shown in bold). (DOCX)

S4 Table Mean values of ecological parameters measured at each study site in Madhya Pradesh. (XLSX)

S5 Table Total species abundance data for each study site in Madhya Pradesh. (XLSX)

S6 Table Mean values of ecological parameters measured at each study site in West Bengal. (XLSX)

S7 Table Total species abundance data for each study site in West Bengal. (XLSX)

S1 Fig Hierarchical cluster analysis of Bray- Curtis distances using complete linkage algorithm of seasonal fish community in West Bengal and Madhya Pradesh show no clear seasonal aggregation. PRM = Pre-monsoon; POM = Post-monsoon; WIN = Winter
(DOCX)

S2 Fig Photographs of some representative fish species collected from West Bengal and Madhya Pradesh. Scientific names of the collected specimen are provided below the photographs.
(DOCX)
