# Supplementary material for: Temporal and environmental drivers of fish-community structure in tropical streams from two contrasting regions in India
Source: PLoS One. 2020 Apr 9;15(4):e0227354. doi: 10.1371/journal.pone.0227354 (PMC7145018; doi:10.1371/journal.pone.0227354)
Supplement: S1 Table — Family-wise list of species in Madhya Pradesh and West Bengal. ‘Pr.’ indicates that the species is present in the region. (DOCX) [file pone.0227354.s001.docx]

**Supplementary Material**

**S1 Table.** Distribution of fish species in Madhya Pradesh and West Bengal. ‘ Pr’ indicates that the species is present in the region. Threat level of all species according to IUCN Red list is provided alongside (LC- least concern; DD- data deficient; NE- not evaluated; NT- near threatened; VU- vulnerable; EN- endangered).

| Family | Species | Madhya Pradesh | West Bengal | IUCN status |
| --- | --- | --- | --- | --- |
| *Cyprinidae* | *Amblypharyngodon mola* | Pr. | Pr. | LC |
|  | *Barilius barila* | - | Pr. | LC |
|  | *Opsarius barna* | Pr. | Pr. | LC |
|  | *Barilius bendelensis* | Pr. | Pr. | LC |
|  | *Barilius modestus* | - | Pr. | LC |
|  | *Barilius shacra* | - | Pr. | LC |
|  | *Barilius vagra* | - | Pr. | LC |
|  | *Chagunius chagunio* | - | Pr. | LC |
|  | *Laubuka laubuca* | Pr. | - | LC |
|  | *Cirrhinus reba* | Pr. | - | LC |
|  | *Tariqilabeo diplochilus* | Pr. | - | NE |
|  | *Devario aequipinnatus* | Pr. | Pr. | LC |
|  | *Danio dangila* | - | Pr. | LC |
|  | *Devario devario* | Pr. | - | LC |
|  | *Danio rerio* | Pr. | Pr. | LC |
|  | *Esomus danrica* | Pr. | Pr. | LC |
|  | *Garra gotyla* | Pr. | - | LC |
|  | *Garra mullya* | Pr. | - | LC |
|  | *Hypselobarbus kurali* | Pr. | - | LC |
|  | *Labeo bata* | Pr. | - | LC |
|  | *Labeo boggut* | Pr. | - | LC |
|  | *Labeo gonius* | Pr. | - | LC |
|  | *Labeo rohita* | Pr. | Pr. | LC |
|  | *Osteobrama cotio* | Pr. | Pr. | LC |
|  | *Puntius amphibius* | Pr. | - | DD |
|  | *Puntius chola* | Pr. | Pr. | LC |
|  | *Pethia conchonius* | - | Pr. | LC |
|  | *Pethia gelius* | Pr. | Pr. | LC |
|  | *Pethia guganio* | Pr. | Pr. | LC |
|  | *Pethia phutonio* | - | Pr. | LC |
|  | *Systomus sarana* | Pr. | Pr. | LC |
|  | *Puntius sophore* | Pr. | Pr. | LC |
|  | *Puntius terio* | Pr. | Pr. | LC |
|  | *Pethia ticto* | Pr. | Pr. | LC |
|  | *Rasbora daniconius* | Pr. | - | LC |
|  | *Salmostoma horai* | Pr. | Pr. | VU |
|  | *Tor khudree* | Pr. | - | EN |
|  | *Tor putitora* | Pr. | - | EN |
|  | *Tor tor* | Pr. | - | DD |
|  |  |  |  |  |
| *Bagridae* | *Mystus bleekeri* | Pr. | Pr. | LC |
|  | *Mystus cavasius* | Pr. | Pr. | LC |
|  | *Mystus tengara* | Pr. | Pr. | LC |
|  | *Mystus vittatus* | Pr. | Pr. | LC |
|  | *Rita gogra* | Pr. | - | LC |
|  | *Rita rita* | Pr. | - | LC |
|  | *Sperata aor* | Pr. | Pr. | LC |
|  |  |  |  |  |
| *Nemacheilidae* | *Paracanthocobitis botia* | Pr. | Pr. | LC |
|  | *Schistura beavani* | Pr. | Pr. | LC |
|  | *Schistura corica* | - | Pr. | LC |
|  | *Schistura denisoni* | Pr. | - | LC |
|  | *Schistura savona* | Pr. | Pr. | LC |
|  |  |  |  |  |
| *Channidae* | *Channa marulius* | Pr. | Pr. | LC |
|  | *Channa gachua* | Pr. | Pr. | LC |
|  | *Channa punctata* | Pr. | Pr. | LC |
|  | *Channa striata* | Pr. | Pr. | LC |
|  |  |  |  |  |
| *Cobitidae* | *Lepidocephalichthys annandelei* | Pr. | Pr. | LC |
|  | *Lepidocephalichthys guntea* | Pr. | Pr. | LC |
|  | *Lepidocephalichthys thermalis* | Pr. | - | LC |
|  | *Pangio pangia* | - | Pr. | LC |
|  | *Somileptes gongota* | - | Pr. | LC |
|  |  |  |  |  |
| *Chacidae* | *Chaca chaca* | - | Pr. | LC |
|  |  |  |  |  |
| *Clariidae* | *Clarias magur* | - | Pr. | EN |
|  |  |  |  |  |
| *Belonidae* | *Xenentodon cancila* | Pr. | Pr. | LC |
|  |  |  |  |  |
| *Erethesitidae* | *Conta conta* | - | Pr. | DD |
|  | *Erethistes jerdoni* | - | Pr. | LC |
|  | *Erethistes hara* | - | Pr. | LC |
|  | *Pseudolaguvia shawi* | Pr. | Pr. | LC |
|  |  |  |  |  |
| *Mastacembelidae* | *Macrognathus aculeatus* | Pr. | Pr. | NE |
|  | *Mastacembelus armatus* | Pr. | Pr. | LC |
|  | *Macrognathus pancalus* | Pr. | Pr. | LC |
|  |  |  |  |  |
| *Heteropneustidae* | *Heteropneustes fossilis* | Pr. | Pr. | LC |
|  |  |  |  |  |
| *Notopteridae* | *Notopterus notopterus* | Pr. | Pr. | LC |
|  | *Chitala chitala* | - | Pr. | LC |
|  |  |  |  |  |
| *Osphronemidae* | *Trichogaster fasciatus* | Pr. | Pr. | LC |
|  | *Trichogaster lalia* | Pr. | Pr. | LC |
|  |  |  |  |  |
| *Psilorhynchidae* | *Psilorhynchus balitora* | - | Pr. | LC |
|  | *Psilorhynchus sucatio* | - | Pr. | LC |
|  |  |  |  |  |
| *Sisoridae* | *Glyptothorax telchitta* | - | Pr. | LC |
|  | *Gagata cenia* | Pr. | - | LC |
|  |  |  |  |  |
| *Synbranchidae* | *Monopterus cuchia* | - | Pr. | LC |
|  |  |  |  |  |
| *Tetraodontidae* | *Leiodon cutcutia* | - | Pr. | LC |
|  |  |  |  |  |
| *Olyridae* | *Olyra longicaudata* | - | Pr. | LC |
|  |  |  |  |  |
| *Gobiidae* | *Glossogobius giuris* | Pr. | Pr. | LC |
|  |  |  |  |  |
| *Ambassidae* | *Chanda nama* | Pr. | Pr. | LC |
|  | *Parambassis lala* | Pr. | Pr. | NT |
|  | *Parambassis ranga* | Pr. | Pr. | LC |
|  |  |  |  |  |
| *Amblycipitidae* | *Amblyceps mangois* | Pr. | Pr. | LC |
|  |  |  |  |  |
| *Badidae* | *Badis badis* | Pr. | Pr. | LC |
|  | *Dario dario* | - | Pr. | DD |
|  |  |  |  |  |
| *Aplocheilidae* | *Aplocheilus panchax* | - | Pr. | LC |
|  |  |  |  |  |
| *Siluridae* | *Ompok bimaculatus* | Pr. | - | NT |
|  | *Wallago attu* | Pr. | - | NT |
|  |  |  |  |  |
| *Mugilidae* | *Chelon parsia* | Pr. | - | NE |
|  |  |  |  |  |
| *Nandidae* | *Nandus nandus* | Pr. | - | LC |
|  |  |  |  |  |
| *Anguillidae* | *Anguilla bengalensis* | - | Pr. | NT |
