# Supplementary material for: Temporal and environmental drivers of fish-community structure in tropical streams from two contrasting regions in India
Source: PLoS One. 2020 Apr 9;15(4):e0227354. doi: 10.1371/journal.pone.0227354 (PMC7145018; doi:10.1371/journal.pone.0227354)
Supplement: S2 Table — Comparisons across seasons, in Species Richness (SR) and Shannon Wiener diversity (H') among seasons in Madhya Pradesh (MP) and West Bengal (WB). Given below are the t-statistic for pairwise comparisons. Values shown in bold are statistically significant. (p-value adjustment method = Bonferroni; p<0.003 was considered statistically significant). (DOCX) [file pone.0227354.s002.docx]

**S2 Table**. **Pairwise comparisons using t-tests ngalh and seasonal variation in rms of diversitys in ng upstrem to test for seasonal variation**. Comparisons across seasons, in a) Species Richness (SR) and b) Shannon Wiener diversity (H') among seasons in Madhya Pradesh (MP) and West Bengal (WB). Given below are the t-statistic for pairwise comparisons. Values shown in bold are statistically significant. (p-value adjustment method= Bonferroni; p<0.003 was considered statistically significant).

a)

|  | MP POM | MP PRM | MP WIN | WB POM | WB PRM |
| --- | --- | --- | --- | --- | --- |
| MP PRM | 2.98 |  |  |  |  |
| MP WIN | 2.22 | **7.53** |  |  |  |
| WB POM | -1.55 | **-3.52** | 0.79 |  |  |
| WB PRM | -0.6 | **-4.49** | 2.43 | 1.1 |  |
| WB WIN | -2.31 | **-4.64** | 0.22 | -0.83 | -1.51 |

b)

|  | MP POM | MP PRM | MP WIN | WB POM | WB PRM |
| --- | --- | --- | --- | --- | --- |
| MP PRM | 1.54 |  |  |  |  |
| MP WIN | -2.77 | **-4.29** |  |  |  |
| WB POM | -1.18 | -3.1 | 1.32 |  |  |
| WB PRM | 0.43 | -2.44 | 3.54 | 1.67 |  |
| WB WIN | -3.56 | **-5.35** | -0.83 | -2.23 | **-3.93** |

(MP WIN= Madhya Pradesh winter; MP PRM= Madhya Pradesh pre monsoon; MP POM= Madhya Pradesh post monsoon; WB WIN= West Bengal winter; WB PRM= West Bengal pre monsoon; WB POM= West Bengal post monsoon)
