# Supplementary material for: Temporal and environmental drivers of fish-community structure in tropical streams from two contrasting regions in India
Source: PLoS One. 2020 Apr 9;15(4):e0227354. doi: 10.1371/journal.pone.0227354 (PMC7145018; doi:10.1371/journal.pone.0227354)
Supplement: S3 Table — (DOCX) [file pone.0227354.s003.docx]

**Supporting Information**

**S3 Table.** Correlation tables showing Pearson’s correlation coefficient (r) and its significance for species richness and diversity in a) Madhya Pradesh and b) West Bengal (p<0.05 are shown in bold).

1. Madhya Pradesh

Species Richness

| Factor | r | p-value |
| --- | --- | --- |
| **Conductivity** | **0.41** | **0.02** |
| **Total dissolved solids** | **0.40** | **0.03** |
| Stream Width | -0.35 | 0.05 |
| Dissolved oxygen.cv | 0.27 | 0.15 |
| Dissolved oxygen | -0.24 | 0.21 |
| Temperature | 0.20 | 0.28 |
| Stream Width.cv | 0.17 | 0.36 |
| pH | 0.10 | 0.59 |
| Water velocity | 0.10 | 0.60 |
| Rainfall | 0.05 | 0.61 |
| Rainfall.cv | -0.10 | 0.61 |
| Altitude | -0.08 | 0.68 |
| Water velocity.cv | -0.06 | 0.73 |
| Total dissolved solids.cv | 0.05 | 0.78 |
| Conductivity.cv | -0.05 | 0.79 |
| Stream depth.cv | -0.05 | 0.81 |
| Temperature.cv | -0.01 | 0.97 |
| Stream depth | 0.00 | 0.99 |
| pH.cv | 0.00 | 1.00 |

Diversity

| Factor | r | p-value |
| --- | --- | --- |
| **Total dissolved solids** | **0.46** | **0.01** |
| **Conductivity** | **0.46** | **0.01** |
| **Dissolved oxygen** | **-0.46** | **0.01** |
| **Temperature** | **0.40** | **0.03** |
| Total dissolved solids.cv | 0.33 | 0.07 |
| Stream Width | -0.33 | 0.07 |
| pH.cv | -0.24 | 0.20 |
| Conductivity.cv | 0.23 | 0.22 |
| Dissolved oxygen.cv | 0.19 | 0.32 |
| Altitude | 0.16 | 0.39 |
| Stream depth.cv | -0.13 | 0.50 |
| Rainfall | 0.12 | 0.58 |
| Rainfall.cv | -0.10 | 0.58 |
| pH | -0.10 | 0.60 |
| Water velocity | -0.09 | 0.65 |
| Stream depth | -0.08 | 0.66 |
| Stream Width.cv | -0.07 | 0.71 |
| Temperature.cv | 0.07 | 0.71 |
| Water velocity.cv | 0.05 | 0.78 |
|  |  |  |

1. West Bengal

Species Richness

| Factor | r | p-value |
| --- | --- | --- |
| **Water velocity** | **-0.39** | **0.03** |
| Conductivity.cv | 0.31 | 0.10 |
| Stream Width | 0.29 | 0.12 |
| Stream depth.cv | -0.29 | 0.12 |
| Altitude | -0.29 | 0.12 |
| Total dissolved solids.cv | 0.26 | 0.16 |
| Temperature | 0.24 | 0.20 |
| Rainfall | 0.09 | 0.22 |
| Rainfall.cv | -0.23 | 0.22 |
| Dissolved oxygen.cv | -0.21 | 0.28 |
| Dissolved oxygen | -0.19 | 0.30 |
| Stream depth | -0.19 | 0.32 |
| Temperature.cv | -0.16 | 0.39 |
| Water velocity.cv | -0.15 | 0.42 |
| Total dissolved solids | -0.08 | 0.66 |
| Conductivity | -0.08 | 0.69 |
| Stream Width.cv | -0.07 | 0.71 |
| pH | -0.03 | 0.87 |
| pH.cv | 0.03 | 0.89 |

Diversity

| Factor | r | p-value |
| --- | --- | --- |
| **Temperature** | **0.44** | **0.01** |
| **Rainfall** | **0.10** | **0.04** |
| **Rainfall.cv** | **-0.38** | **0.04** |
| **Water velocity** | **-0.37** | **0.04** |
| Total dissolved solids | -0.35 | 0.06 |
| Conductivity | -0.32 | 0.09 |
| Dissolved oxygen | -0.32 | 0.09 |
| Water velocity.cv | -0.28 | 0.13 |
| pH.cv | -0.23 | 0.22 |
| Stream depth.cv | -0.23 | 0.22 |
| Stream Width.cv | -0.17 | 0.37 |
| Stream Width | -0.15 | 0.45 |
| pH | -0.14 | 0.47 |
| Total dissolved solids.cv | -0.13 | 0.49 |
| Stream depth | 0.12 | 0.53 |
| Altitude | -0.06 | 0.77 |
| Conductivity.cv | -0.05 | 0.80 |
| Dissolved oxygen.cv | -0.03 | 0.86 |
| Temperature.cv | -0.01 | 0.97 |
