# Supplementary material for: Temporal and environmental drivers of fish-community structure in tropical streams from two contrasting regions in India
Source: PLoS One. 2020 Apr 9;15(4):e0227354. doi: 10.1371/journal.pone.0227354 (PMC7145018; doi:10.1371/journal.pone.0227354)

**S1 Figure.** Hierarchical cluster analysis of Bray- Curtis distances using complete linkage algorithm of seasonal fish community in a) West Bengal and b) Madhya Pradesh show no clear seasonal aggregation. PRM= Pre-monsoon; POM=Post-monsoon; WIN=Winter

a)


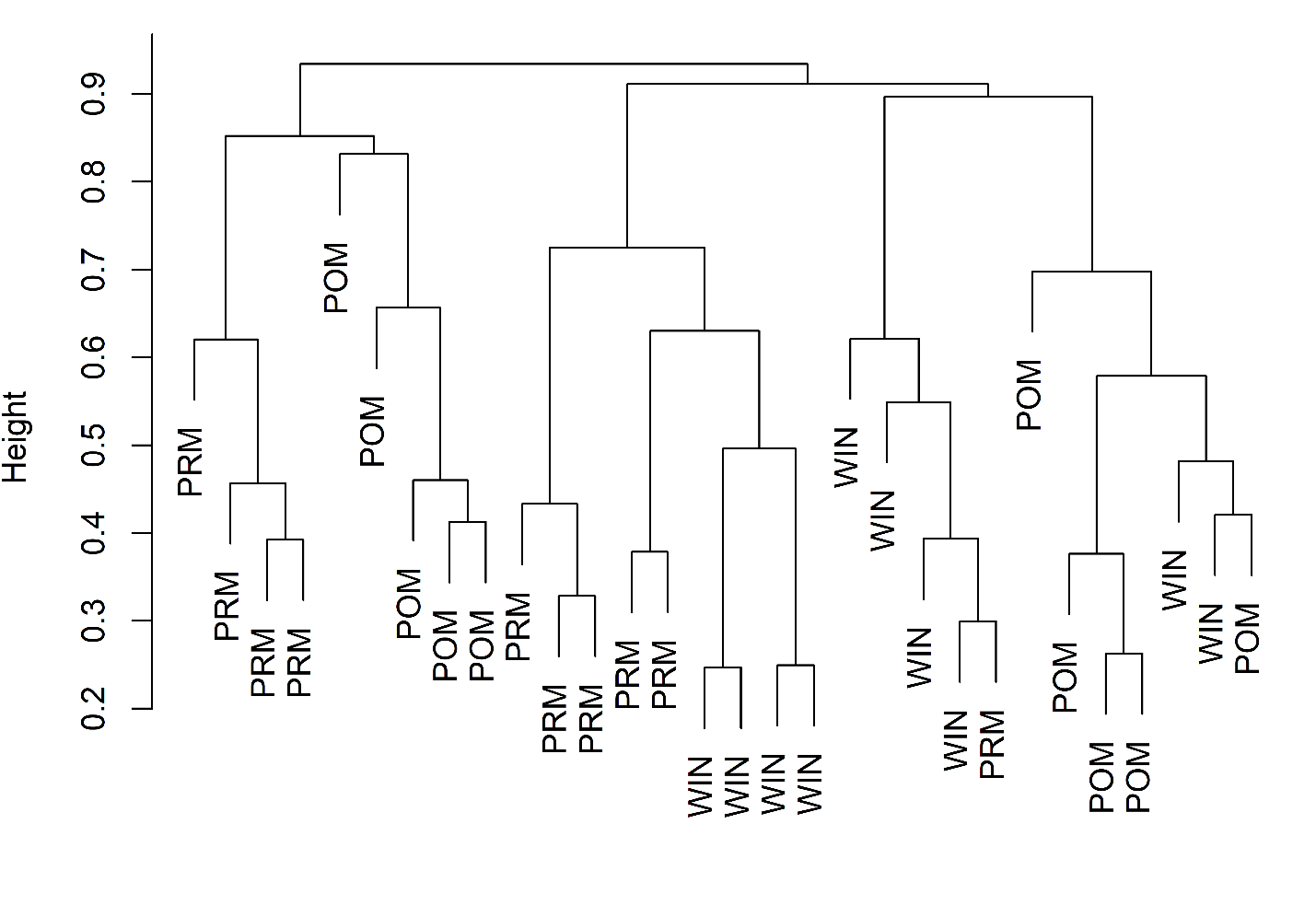


b)


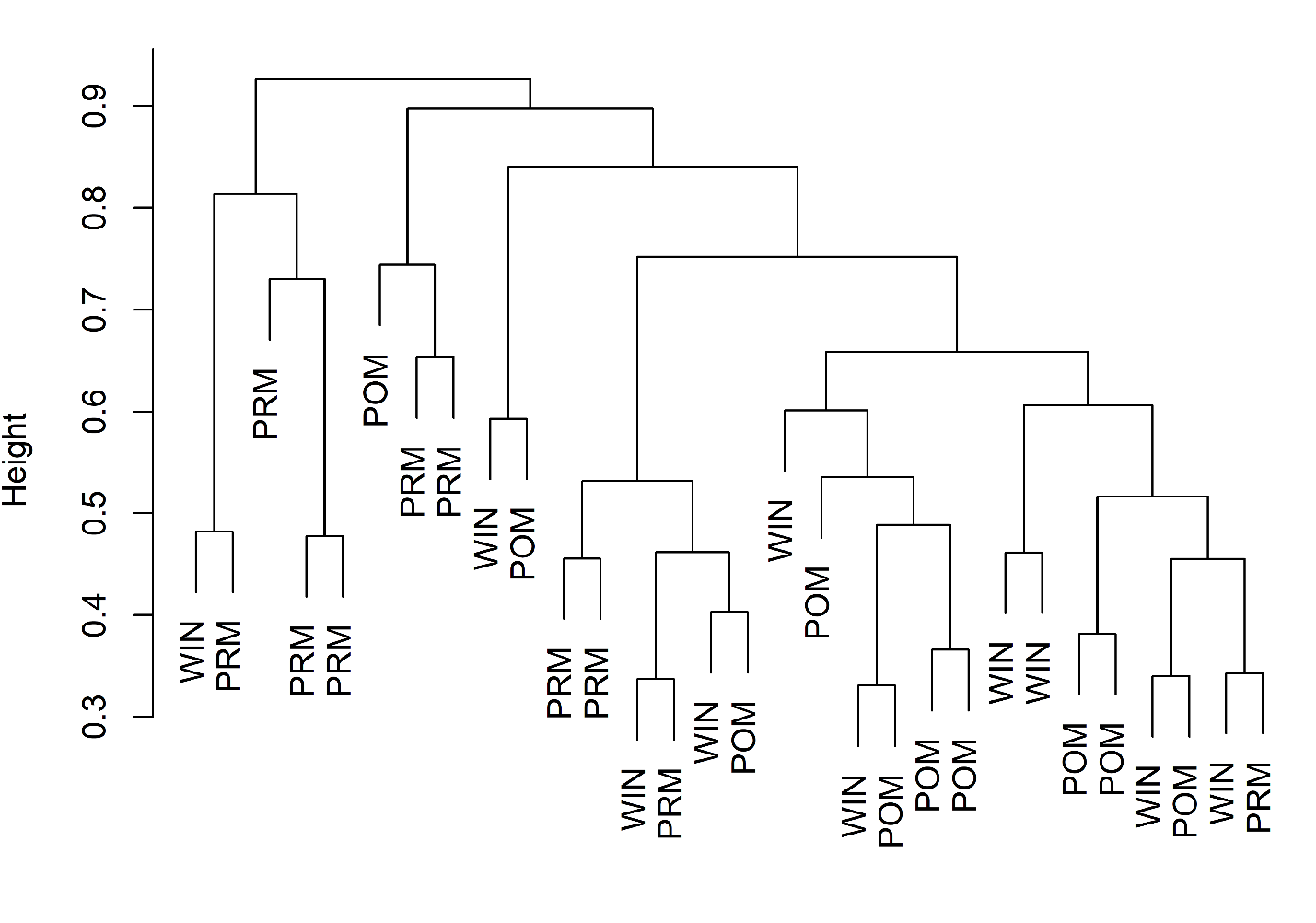

Supplement: S1 Fig — PRM = Pre-monsoon; POM = Post-monsoon; WIN = Winter (DOCX) [file pone.0227354.s008.docx]
