# Supplementary material for: Temporal and environmental drivers of fish-community structure in tropical streams from two contrasting regions in India
Source: PLoS One. 2020 Apr 9;15(4):e0227354. doi: 10.1371/journal.pone.0227354 (PMC7145018; doi:10.1371/journal.pone.0227354)
Supplement: S2 Fig — Scientific names of the collected specimen are provided below the photographs. (DOCX) [file pone.0227354.s009.docx]

**S2 Figure. Photographs of some representative fish species collected from West Bengal and Madhya Pradesh.** Scientific names of each collected specimen are provided at the bottom of each photograph.

1 cm


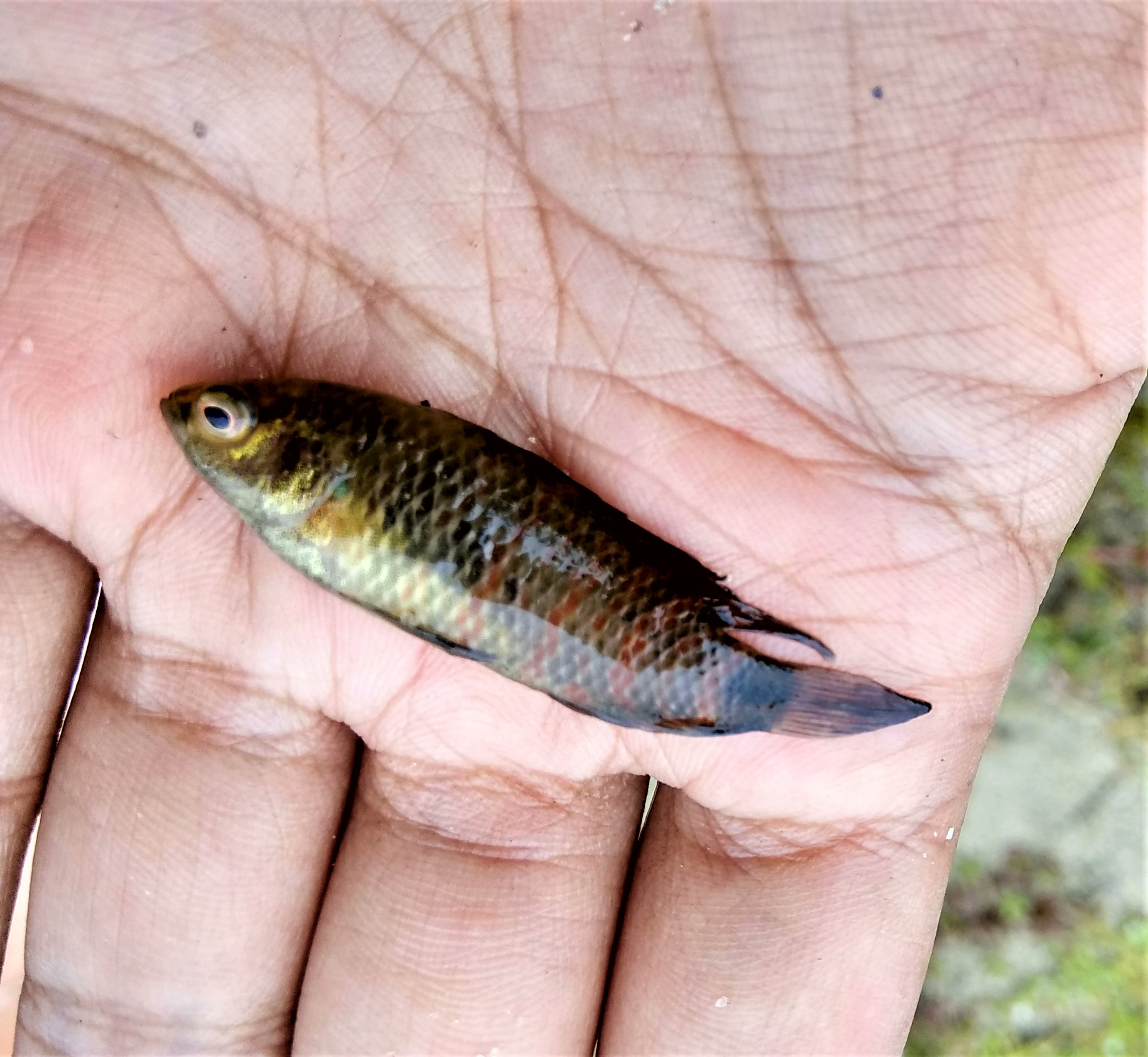


*Badis badis* (Hamilton, 1822)

1 cm


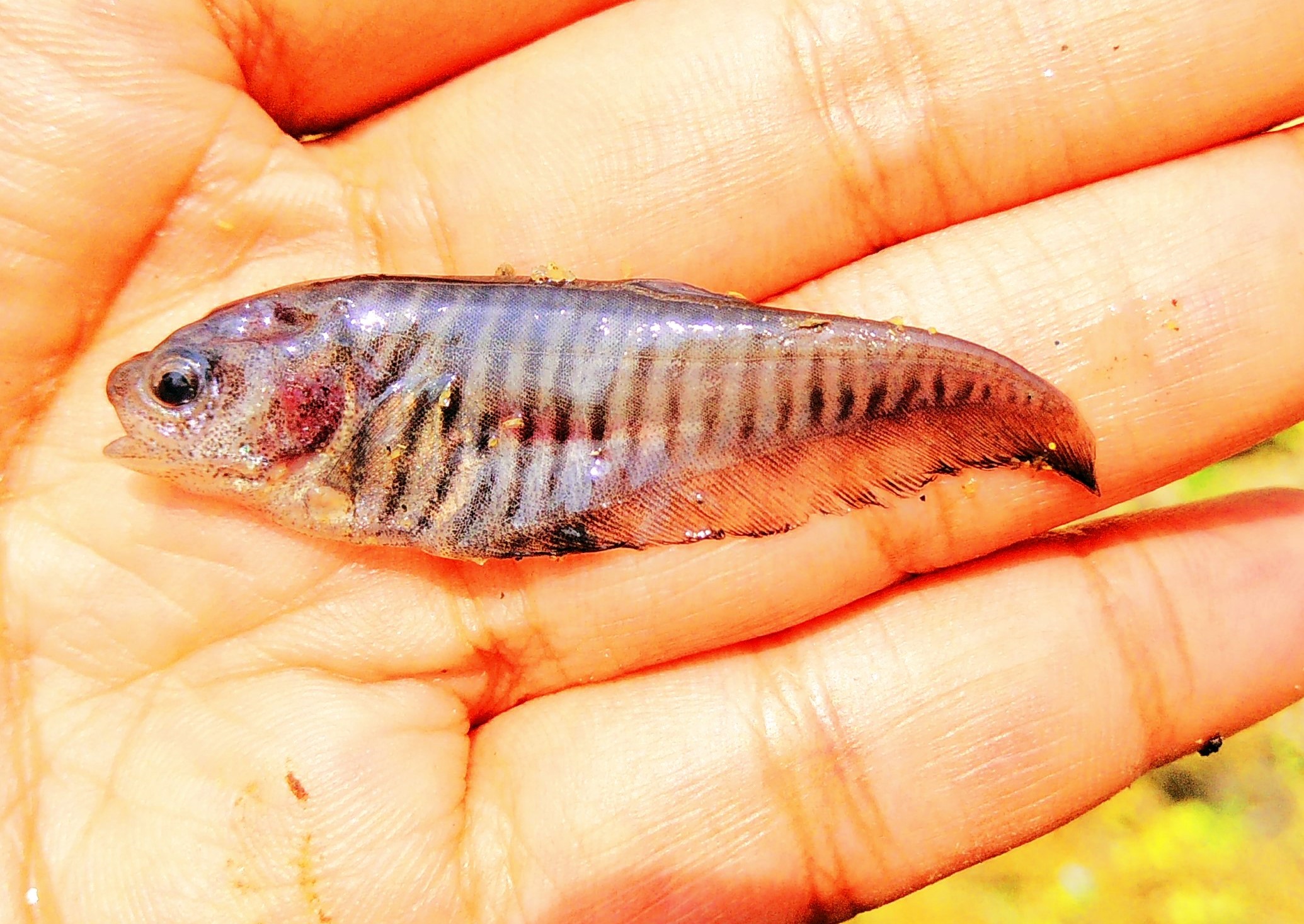


*Chitala chitala* (Hamilton, 1822)

1 cm


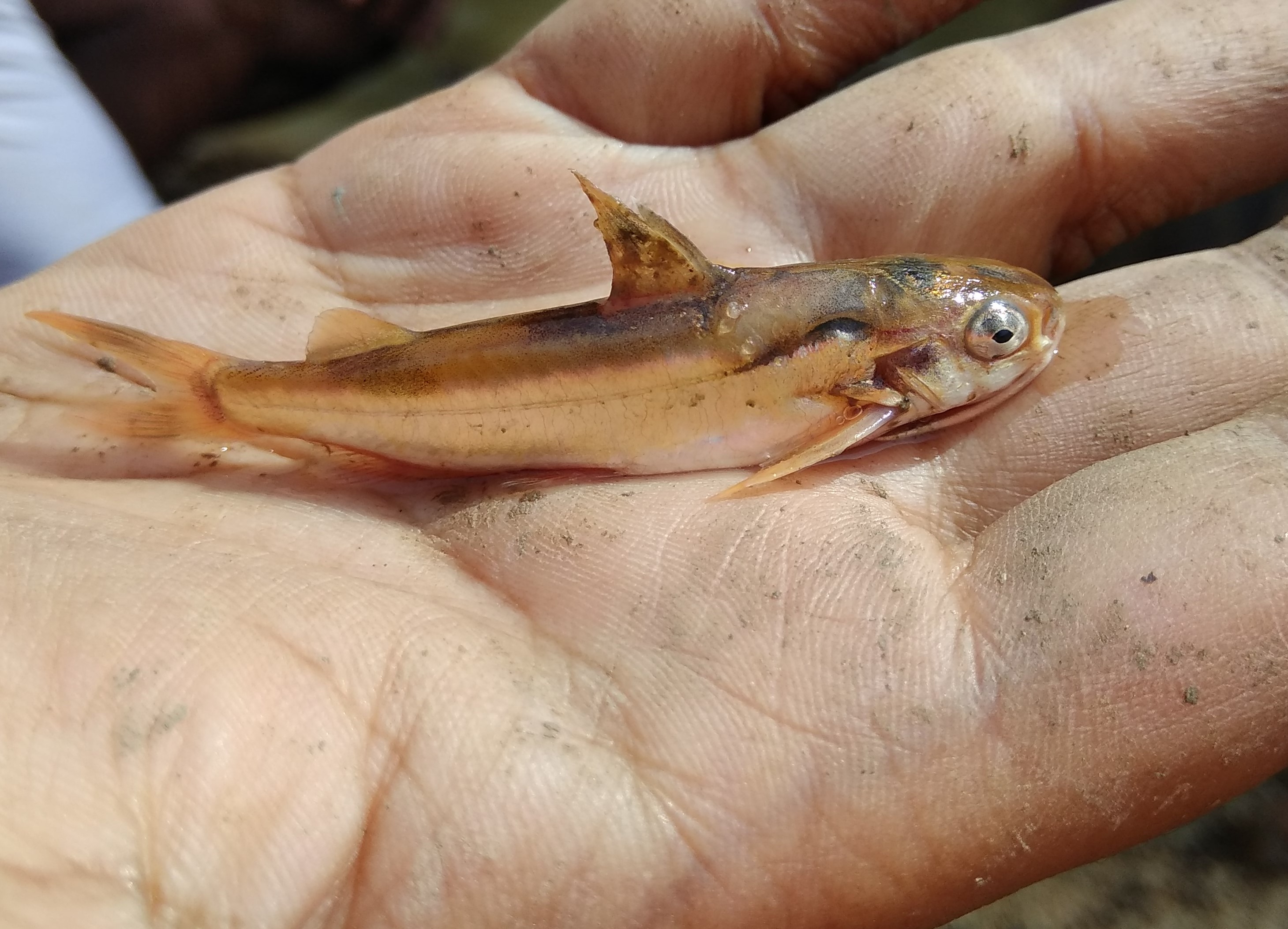


*Gagata cenia* (Hamilton, 1822)

1 cm


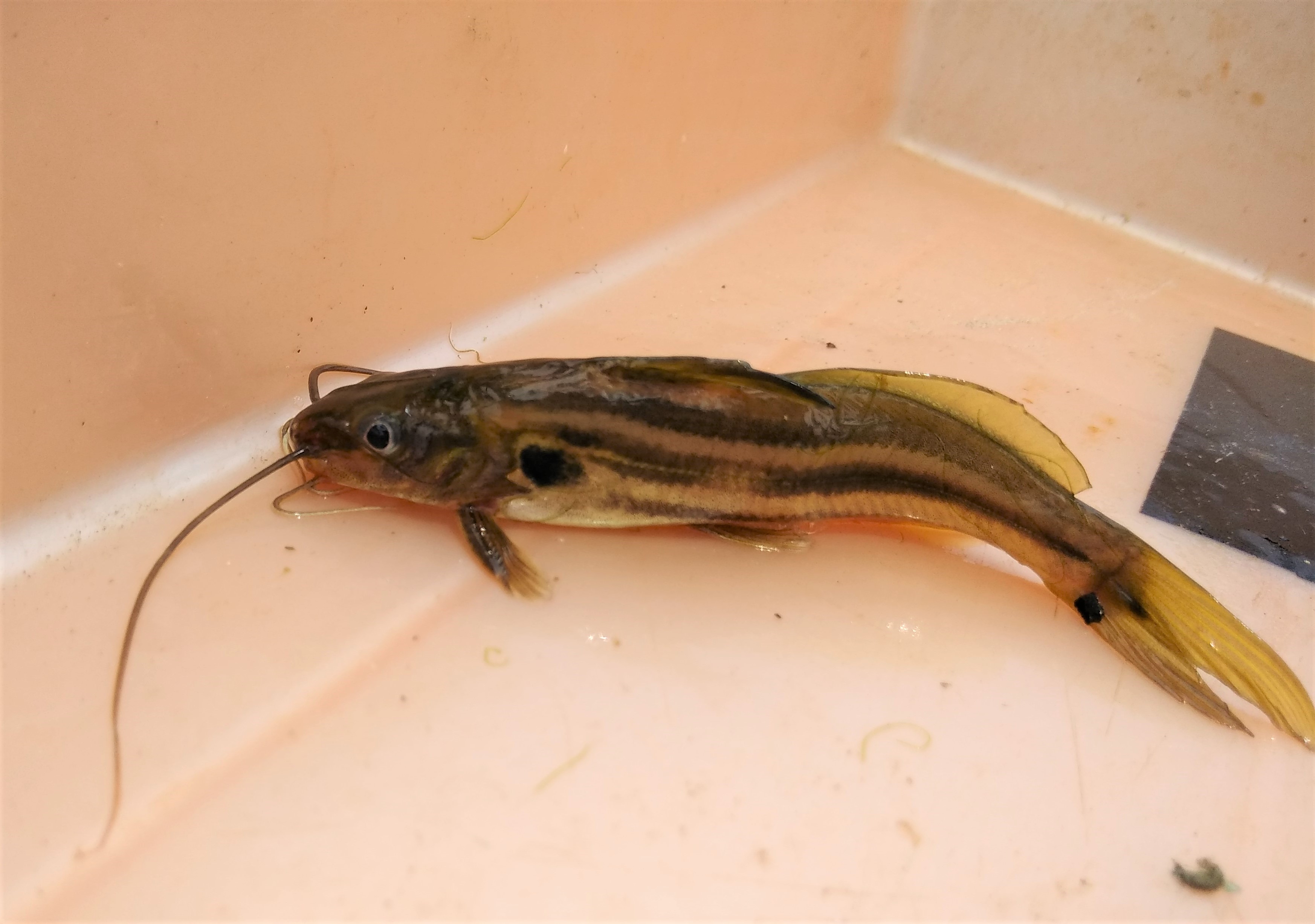


*Mystus bleekeri* (Day, 1877)

1 cm


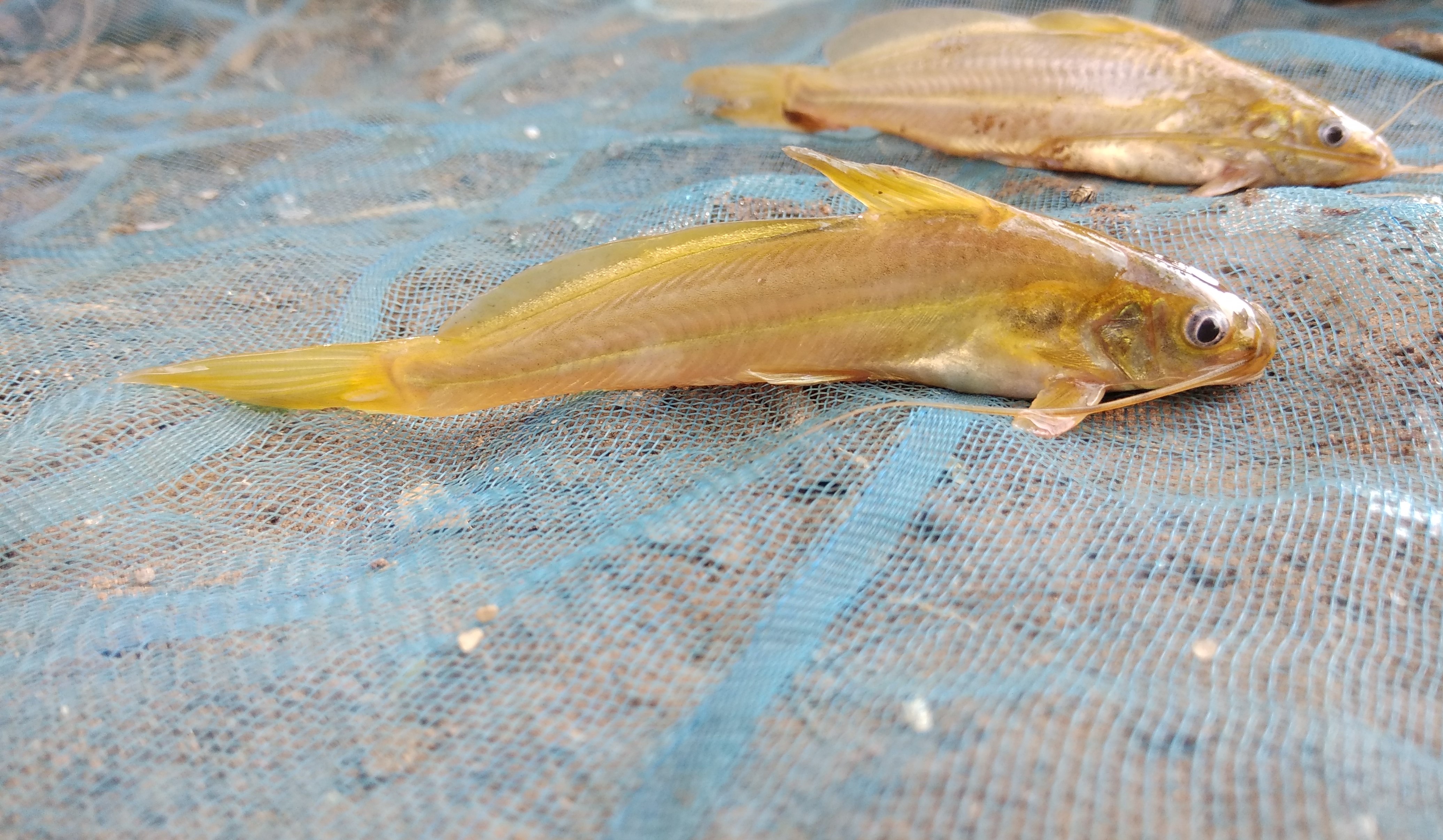


*Mystus cavasius* (Hamilton, 1822)

1 cm


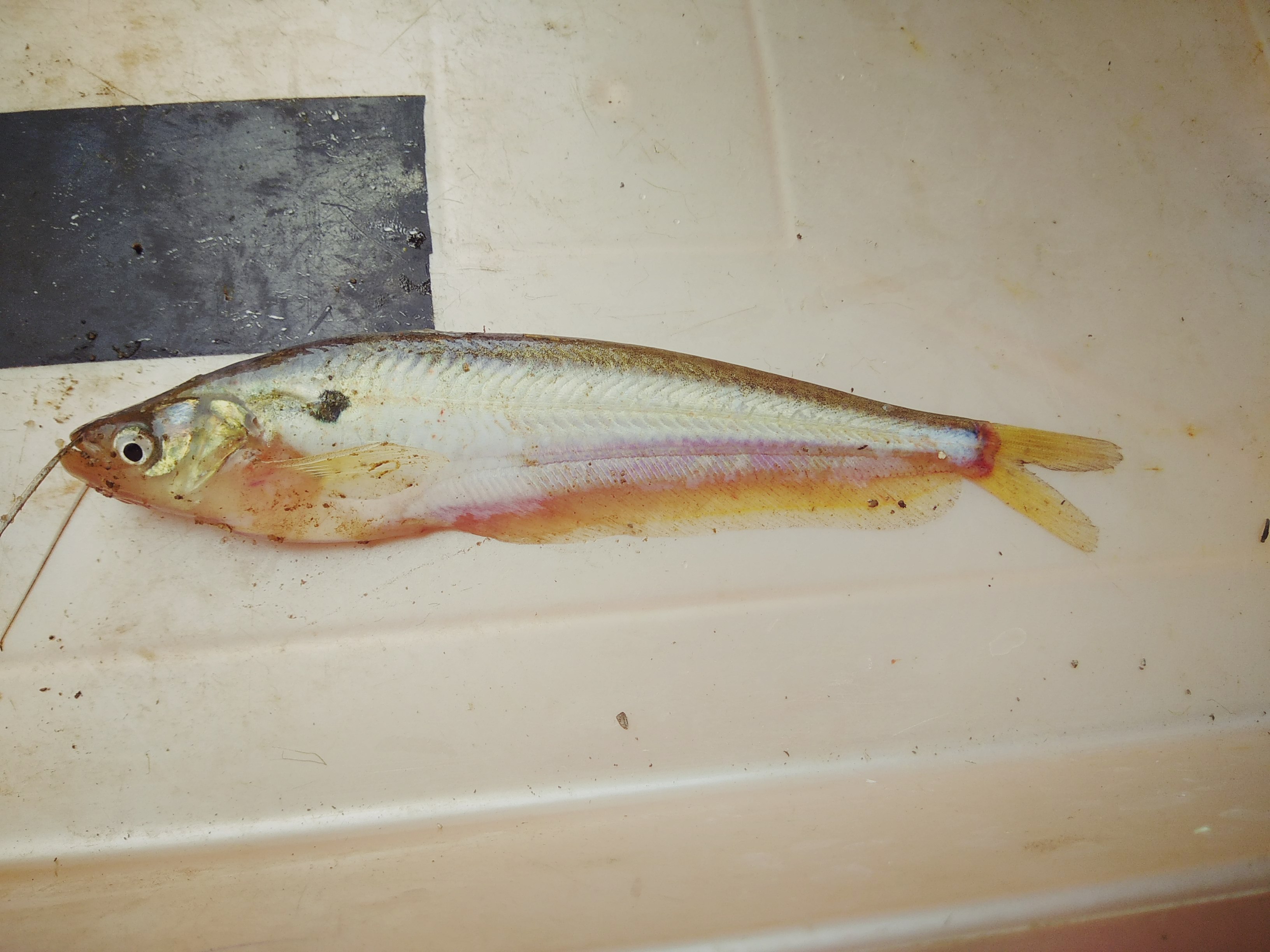


*Ompok bimaculatus* (Bloch, 1794)

1 cm


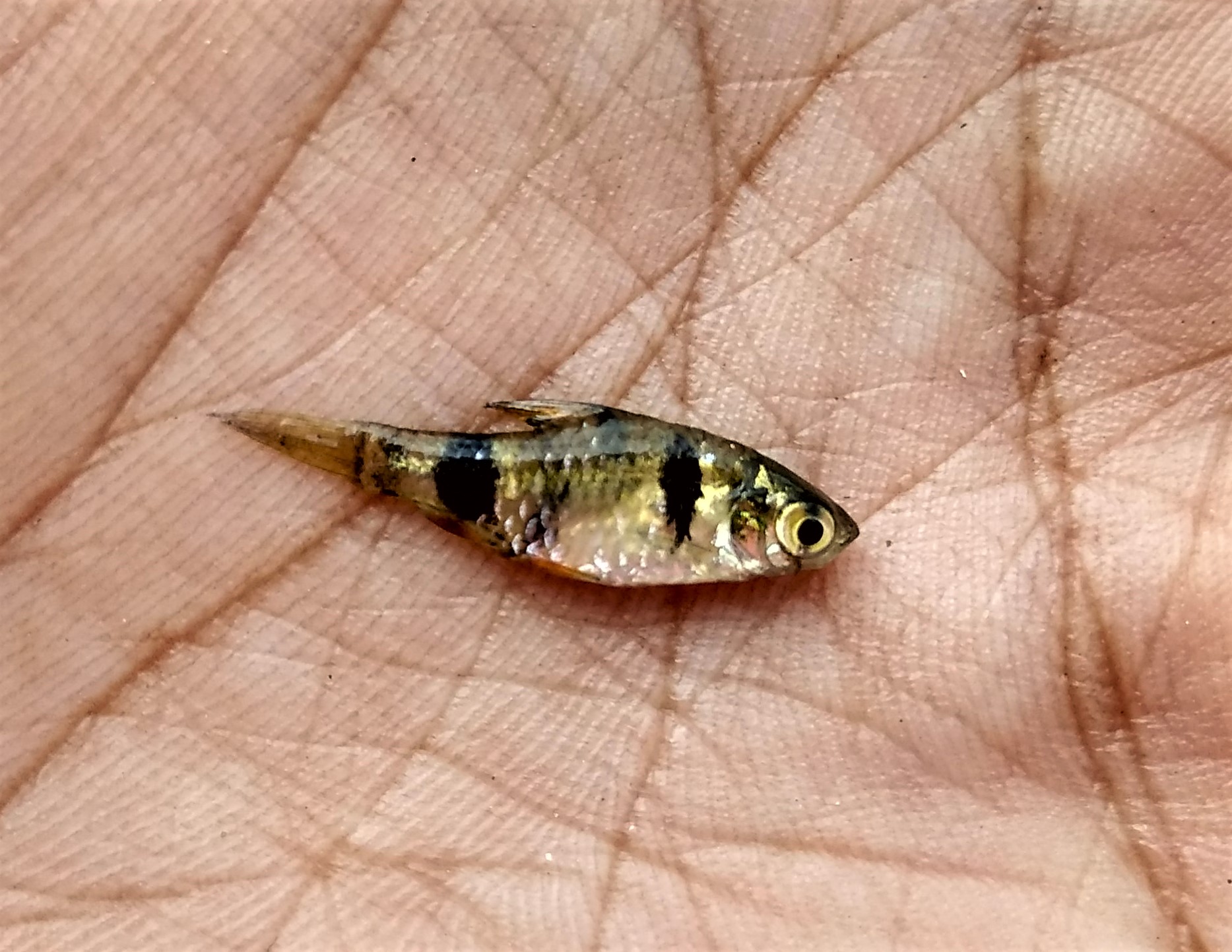


*Pethia gelius* (Hamilton, 1822)

1 cm


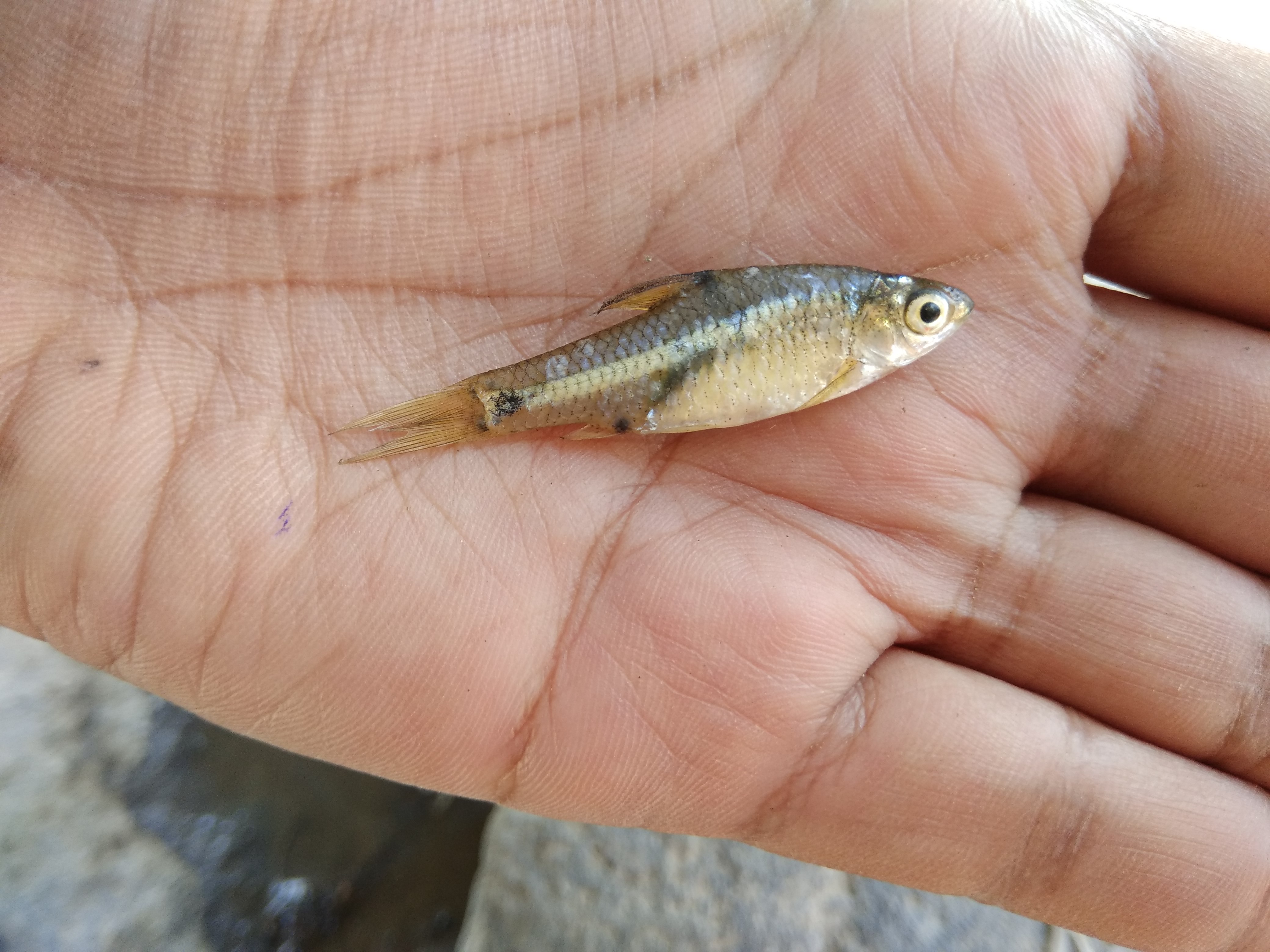


*Pethia guganio* (Hamilton, 1822)

1 cm


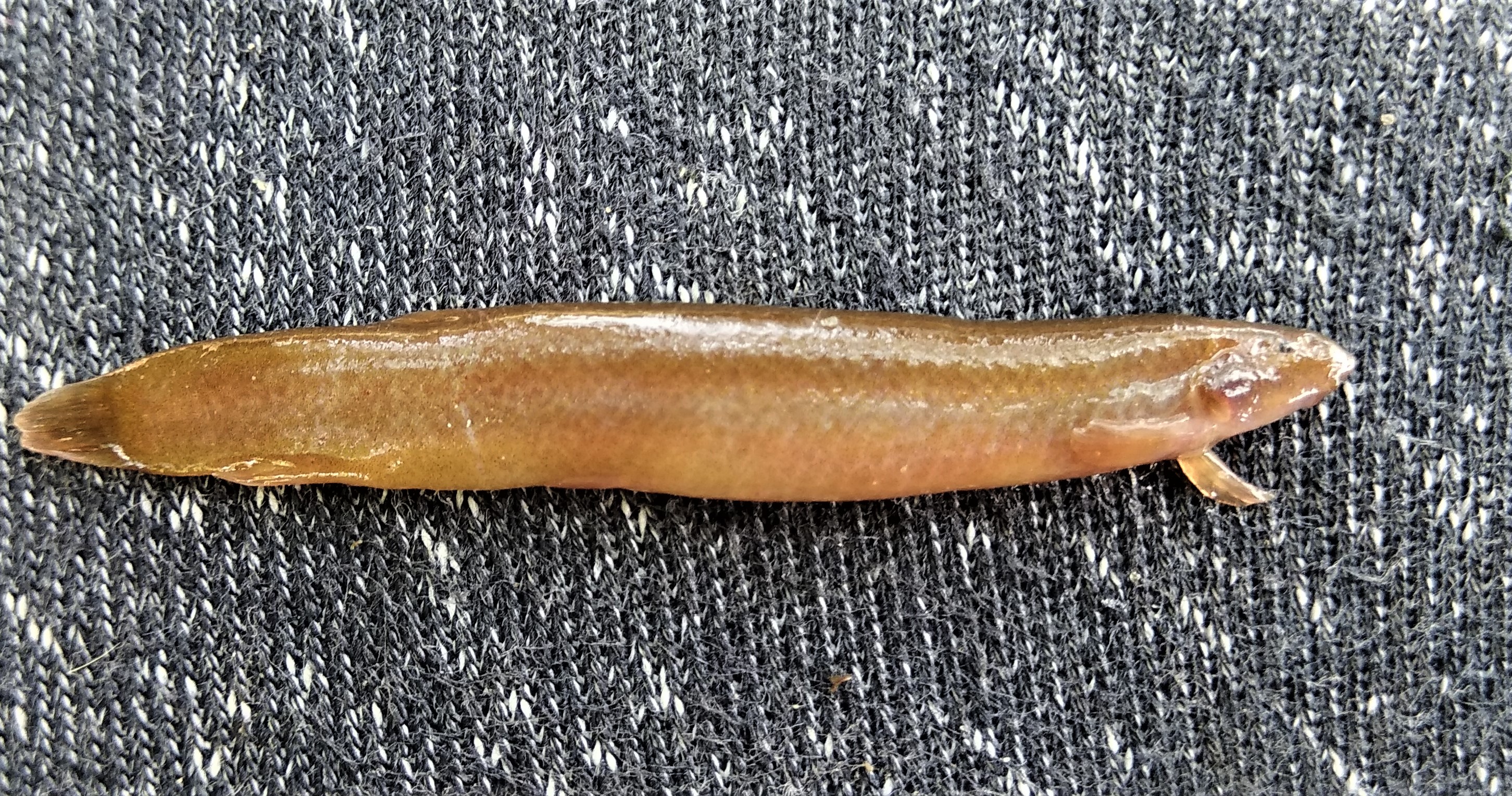


*Pangio pangia* (Hamilton, 1822)

1 cm


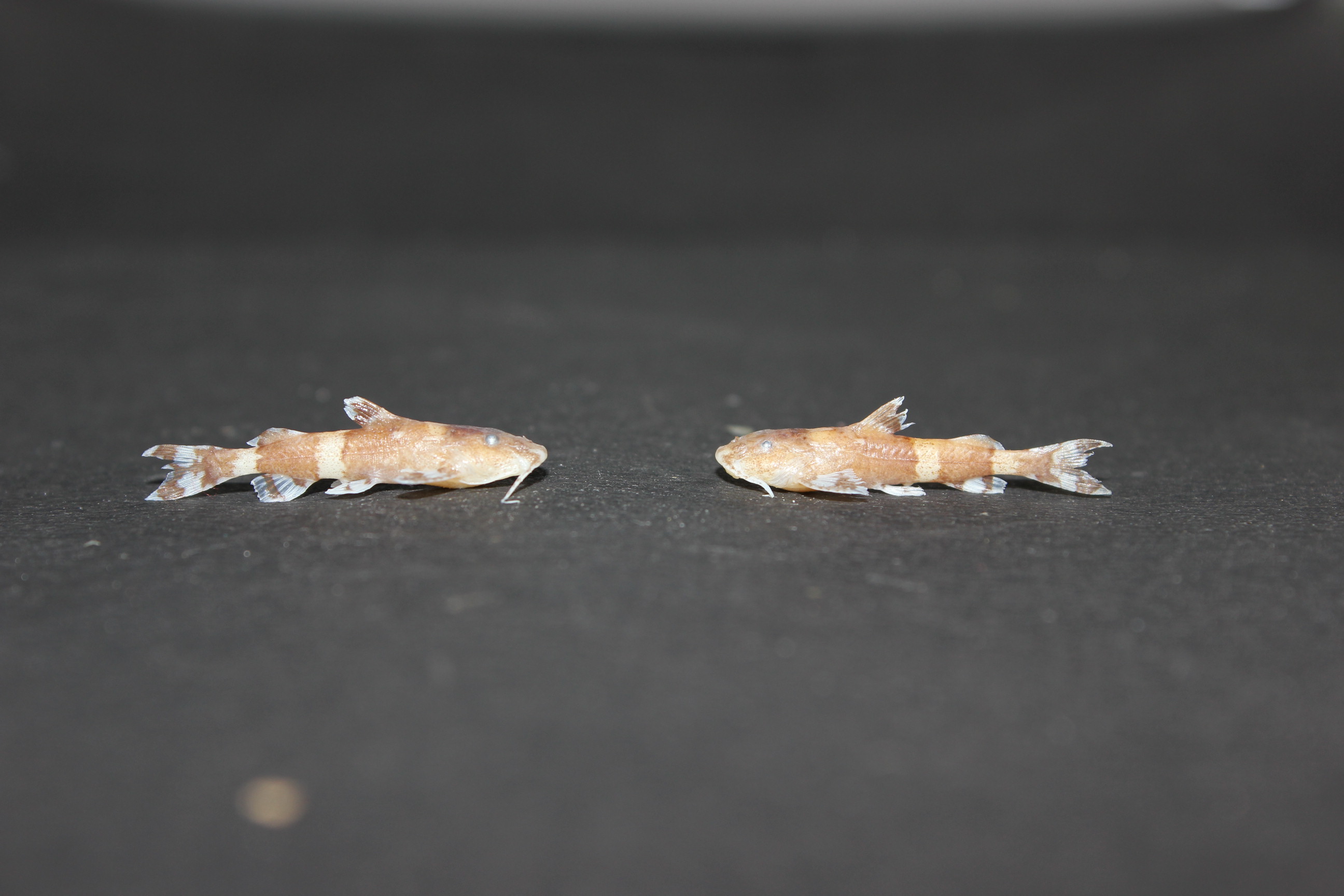


*Pseudolaguvia shawi* (Hora, 1921)

1 cm


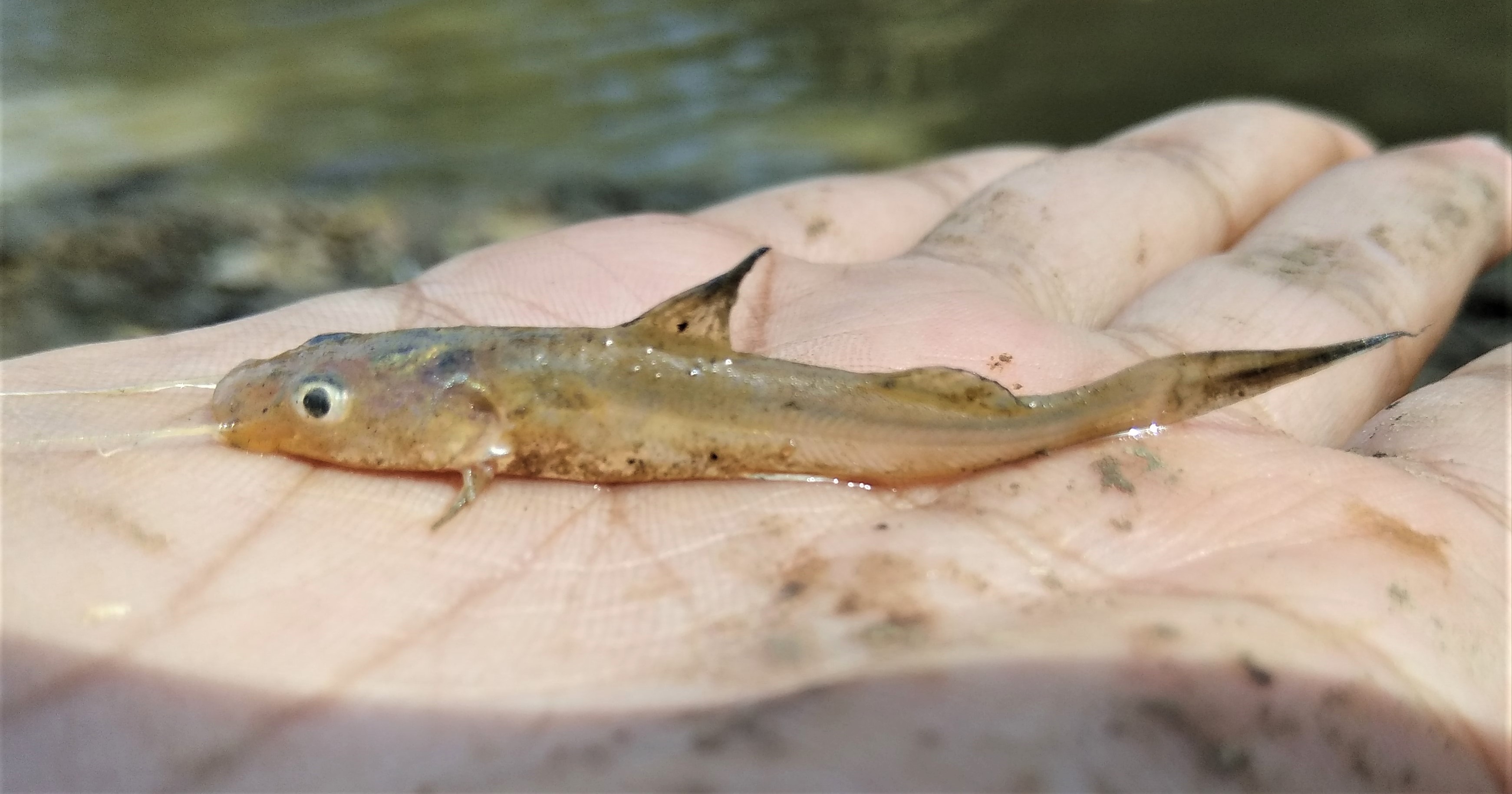


*Sperata aor* (Hamilton, 1822)

1 cm


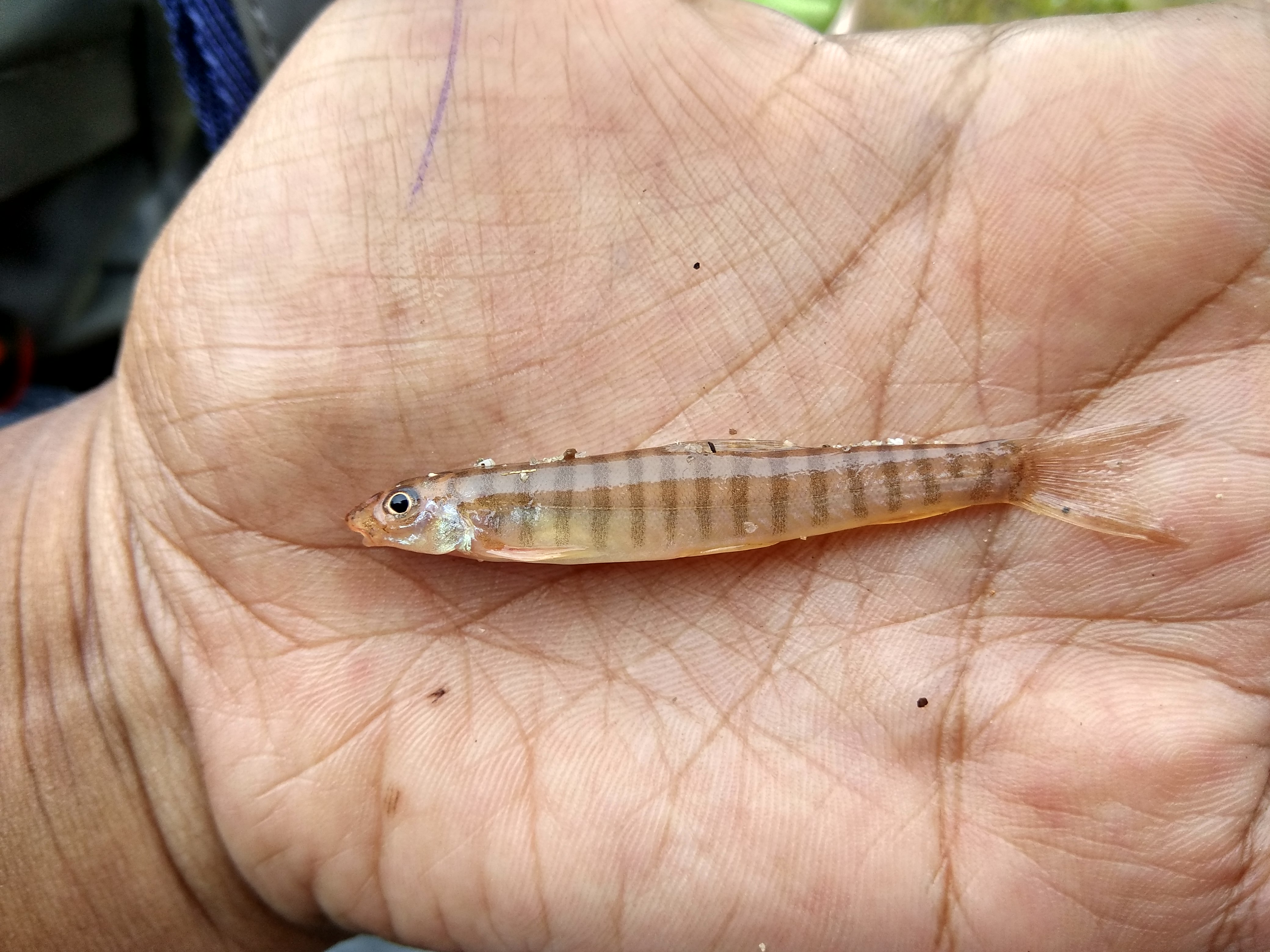


*Schistura beavani* (Günther, 1868)

1 cm


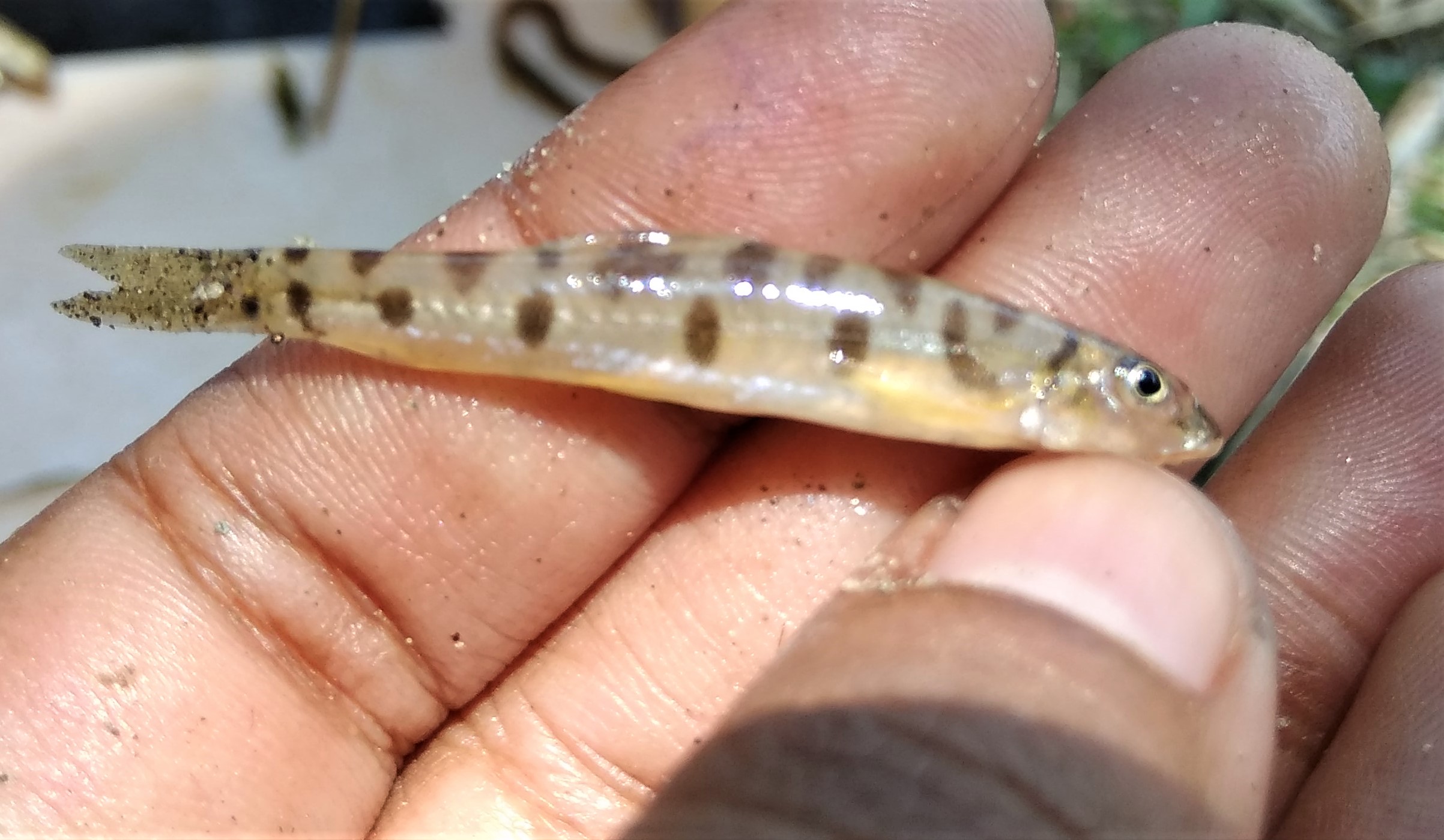


*Schistura corica* (Hamilton, 1822)

1 cm


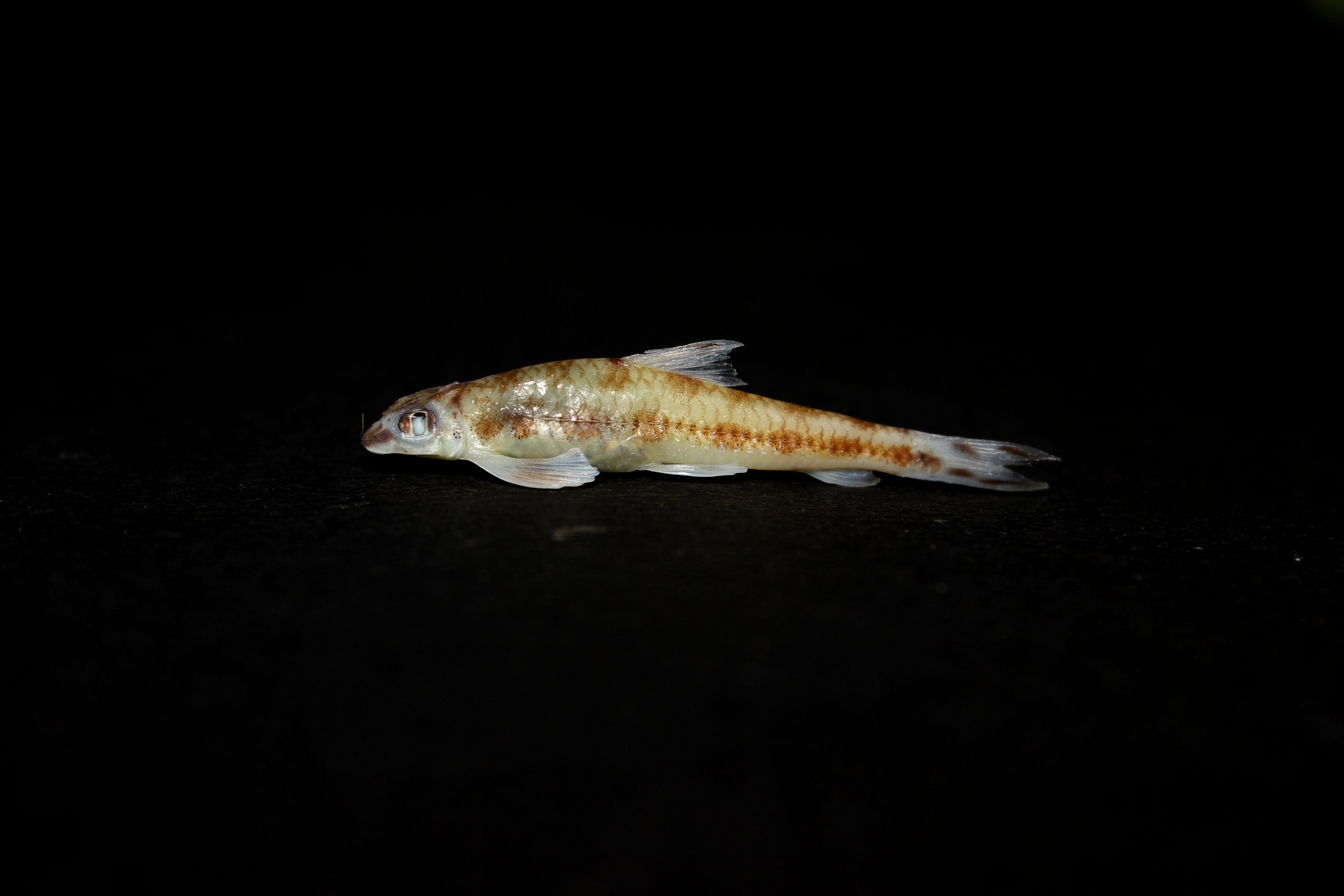


*Psilorhynchus balitora* (Hamilton, 1822)

1 cm


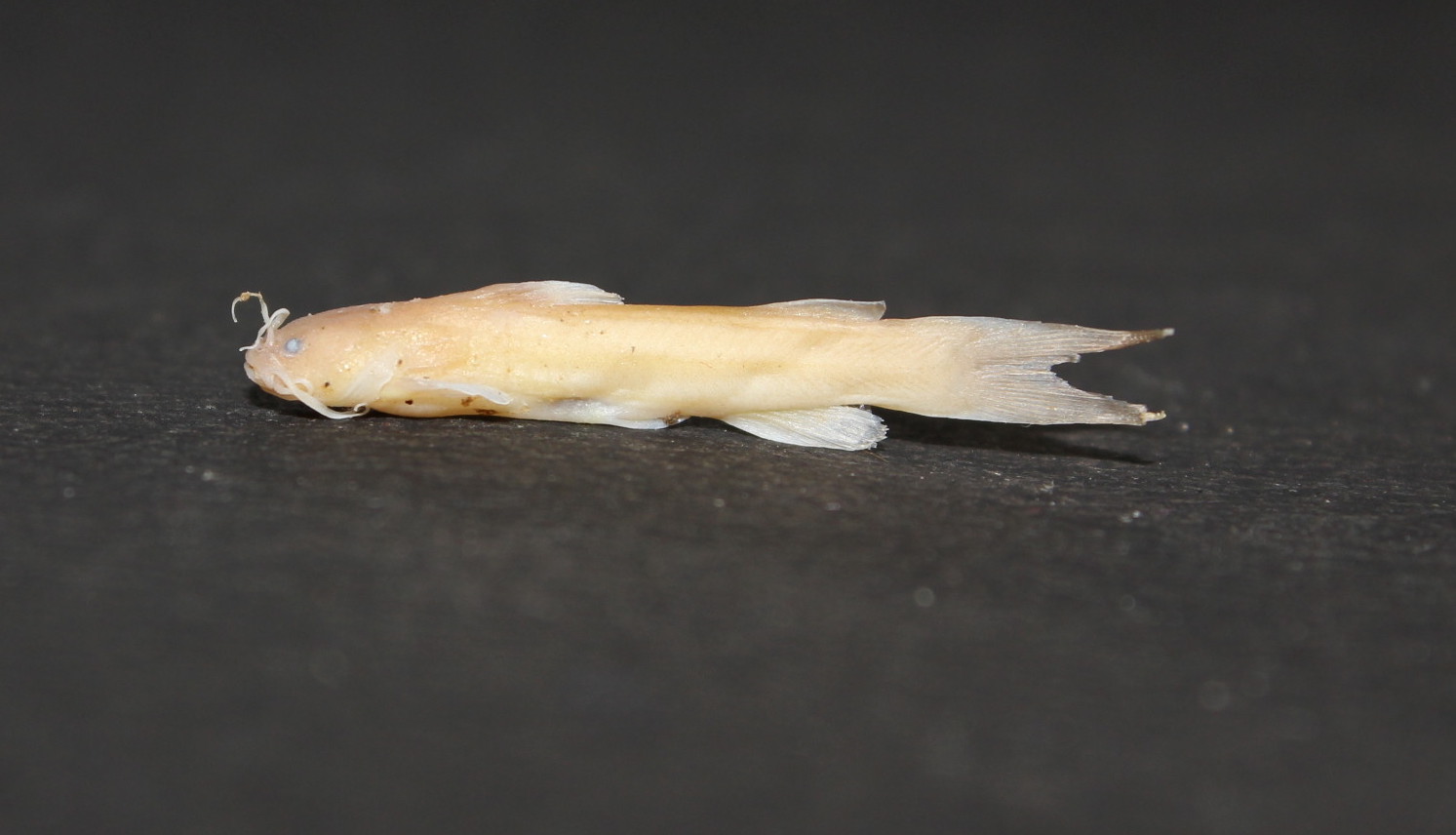


*Ambyceps mangois* (Hamilton, 1822)

1 cm


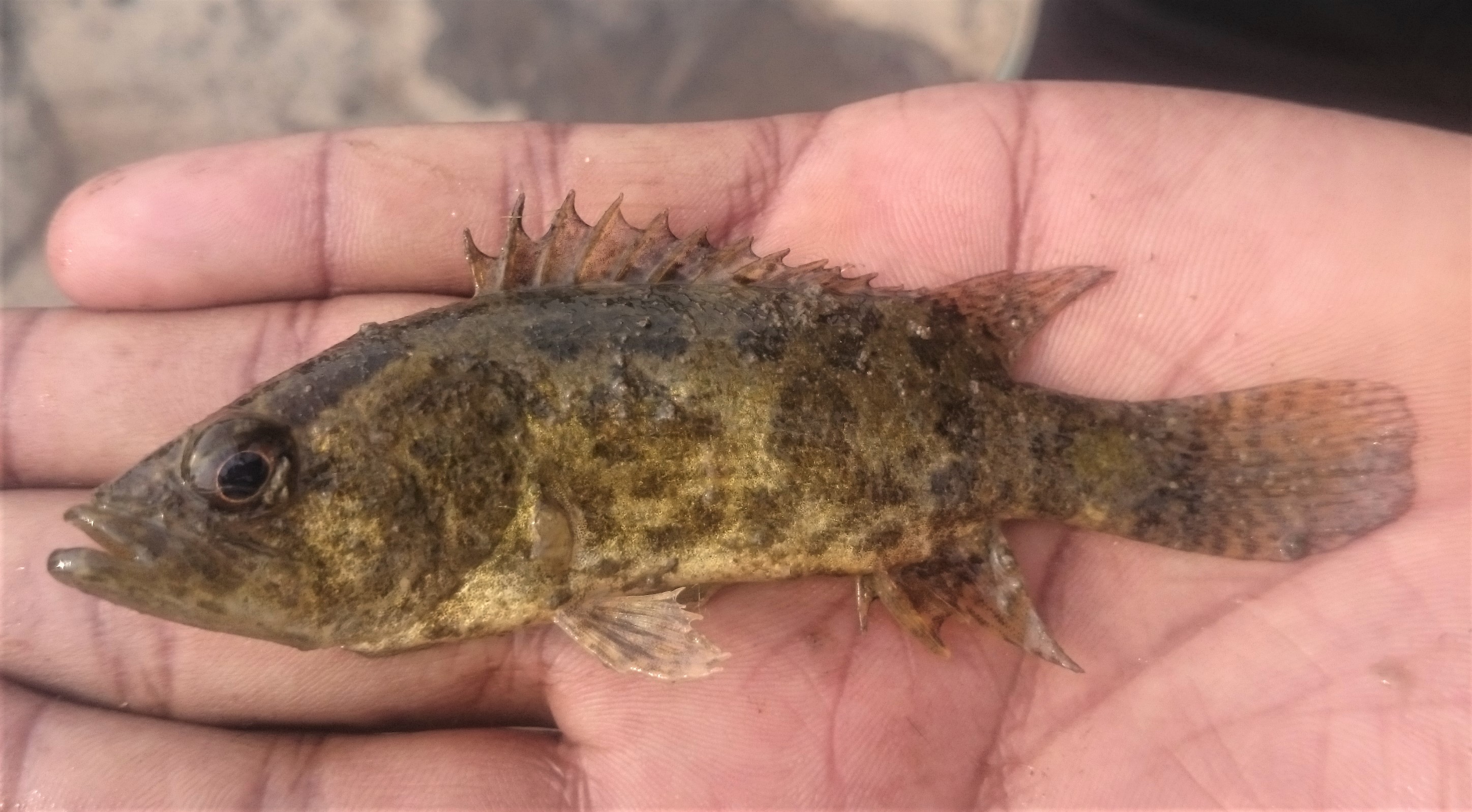


*Nandus nandus* (Hamilton, 1822)

*
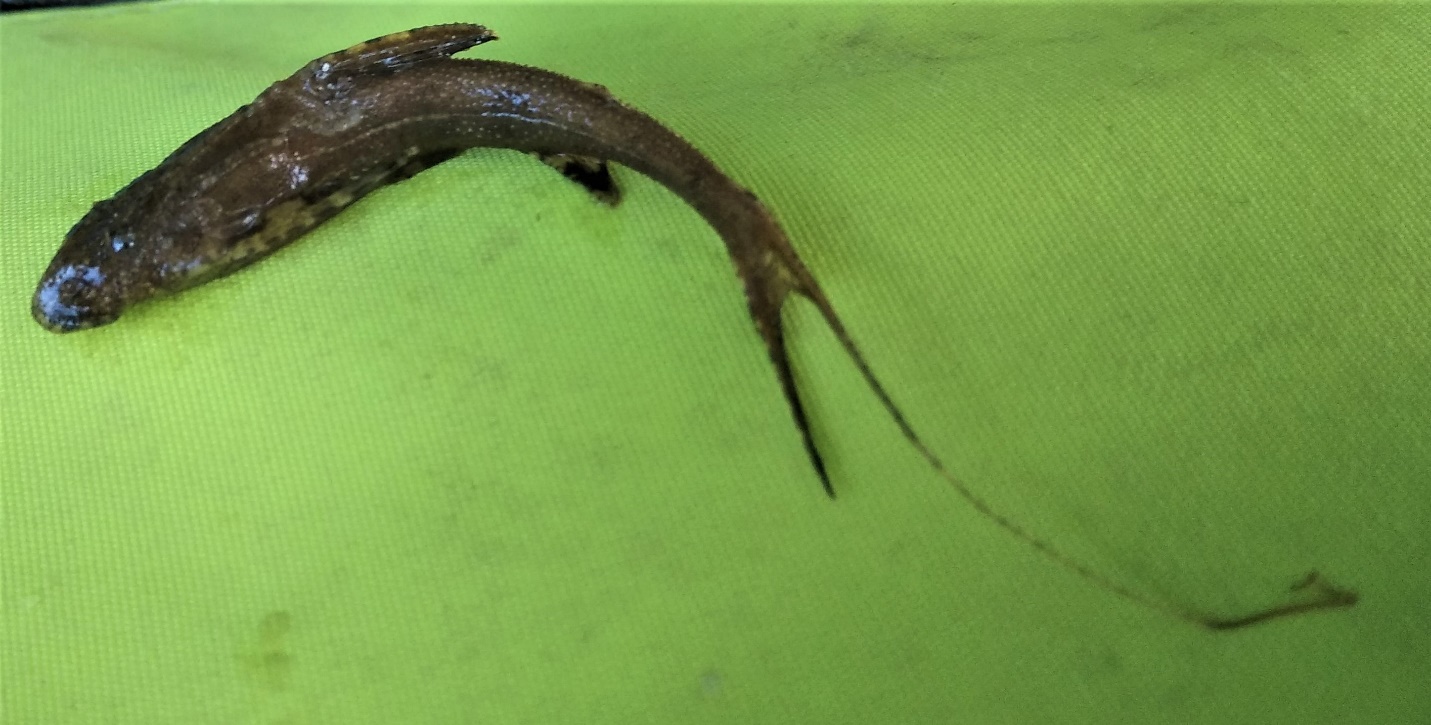
*

1 cm

*Olyra longicaudata* (McClelland, 1842)

1 cm


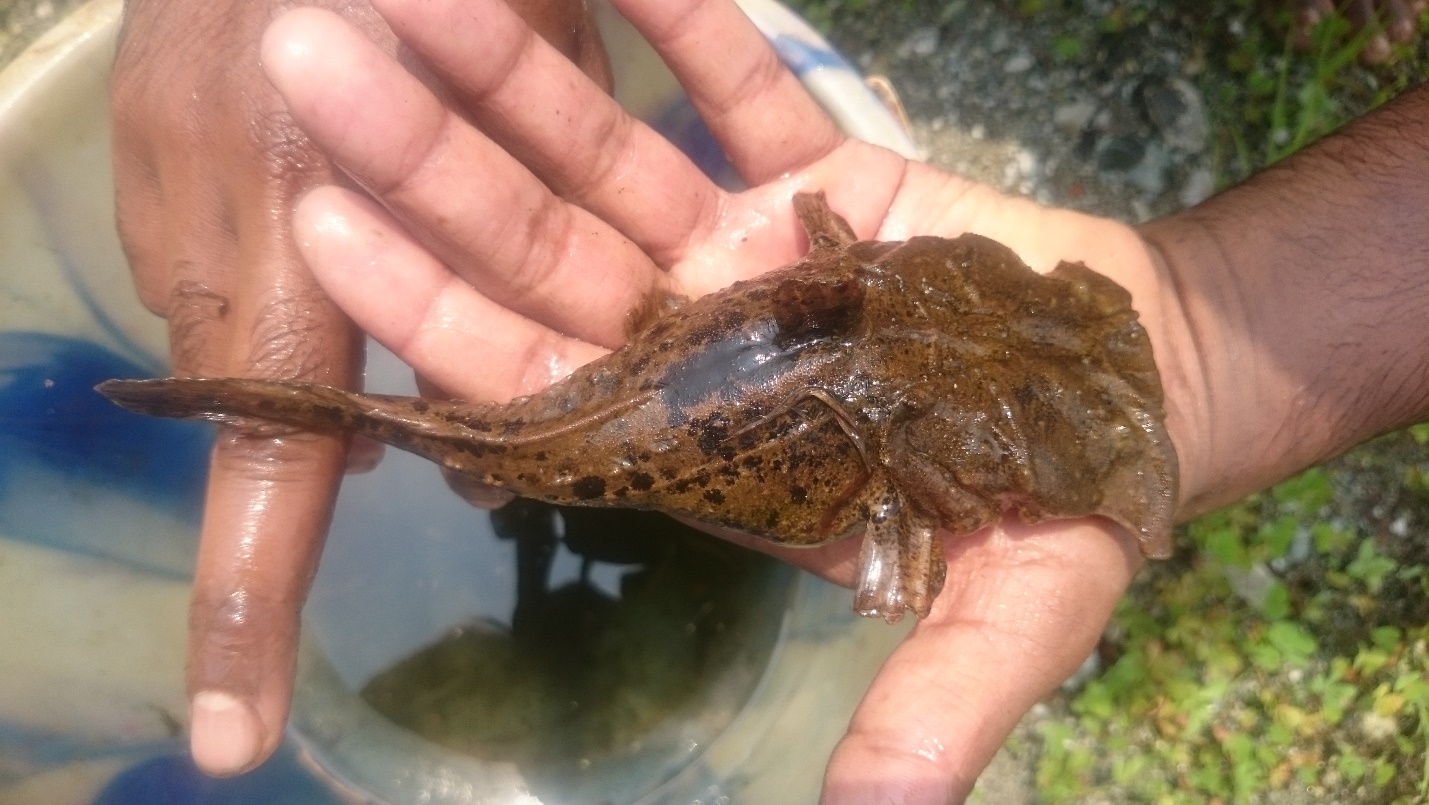
 *Chaca chaca* (Hamilton, 1822)
